# Substrate Recognition by the Peptidyl-(*S*)-2-mercaptoglycine Synthase TglHI during 3-Thiaglutamate Biosynthesis

**DOI:** 10.1021/acschembio.2c00087

**Published:** 2022-04-01

**Authors:** Martin I. McLaughlin, Yue Yu, Wilfred A. van der Donk

**Affiliations:** † Department of Chemistry and Carl R. Woese Institute for Genomic Biology, University of Illinois at Urbana-Champaign, Urbana, Illinois 61801, United States; ‡ Howard Hughes Medical Institute, University of Illinois at Urbana-Champaign, Urbana, Illinois 61801, United States

## Abstract

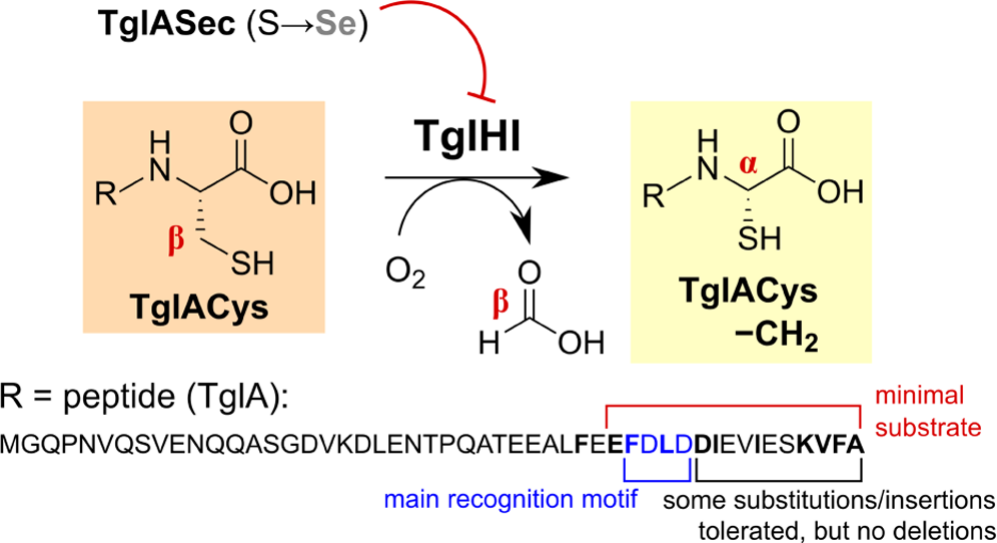

3-Thiaglutamate is a recently identified amino acid analog originating from cysteine. During its biosynthesis, cysteinyl-tRNA is first enzymatically appended to the C-terminus of TglA, a 50-residue ribosomally translated peptide scaffold. After hydrolytic removal of the tRNA, this cysteine residue undergoes modification on the scaffold before eventual proteolysis of the nascent 3-thiaglutamyl residue to release 3-thiaglutamate and regenerate TglA. One of the modifications of TglACys requires a complex of two polypeptides, TglH and TglI, which uses nonheme iron and O_2_ to catalyze the removal of the peptidyl-cysteine β-methylene group, oxidation of this Cβ atom to formate, and reattachment of the thiol group to the α carbon. Herein, we use *in vitro* transcription-coupled translation and expressed protein ligation to characterize the role of the TglA scaffold in TglHI recognition and determine the specificity of TglHI with respect to the C-terminal residues of its substrate TglACys. The results of these experiments establish a synthetically accessible TglACys fragment sufficient for modification by TglHI and identify the l-selenocysteine analog of TglACys, TglASec, as an inhibitor of TglHI. These insights as well as a predicted structure and native mass spectrometry data set the stage for deeper mechanistic investigation of the complex TglHI-catalyzed reaction.

## Introduction

**Figure 1:**
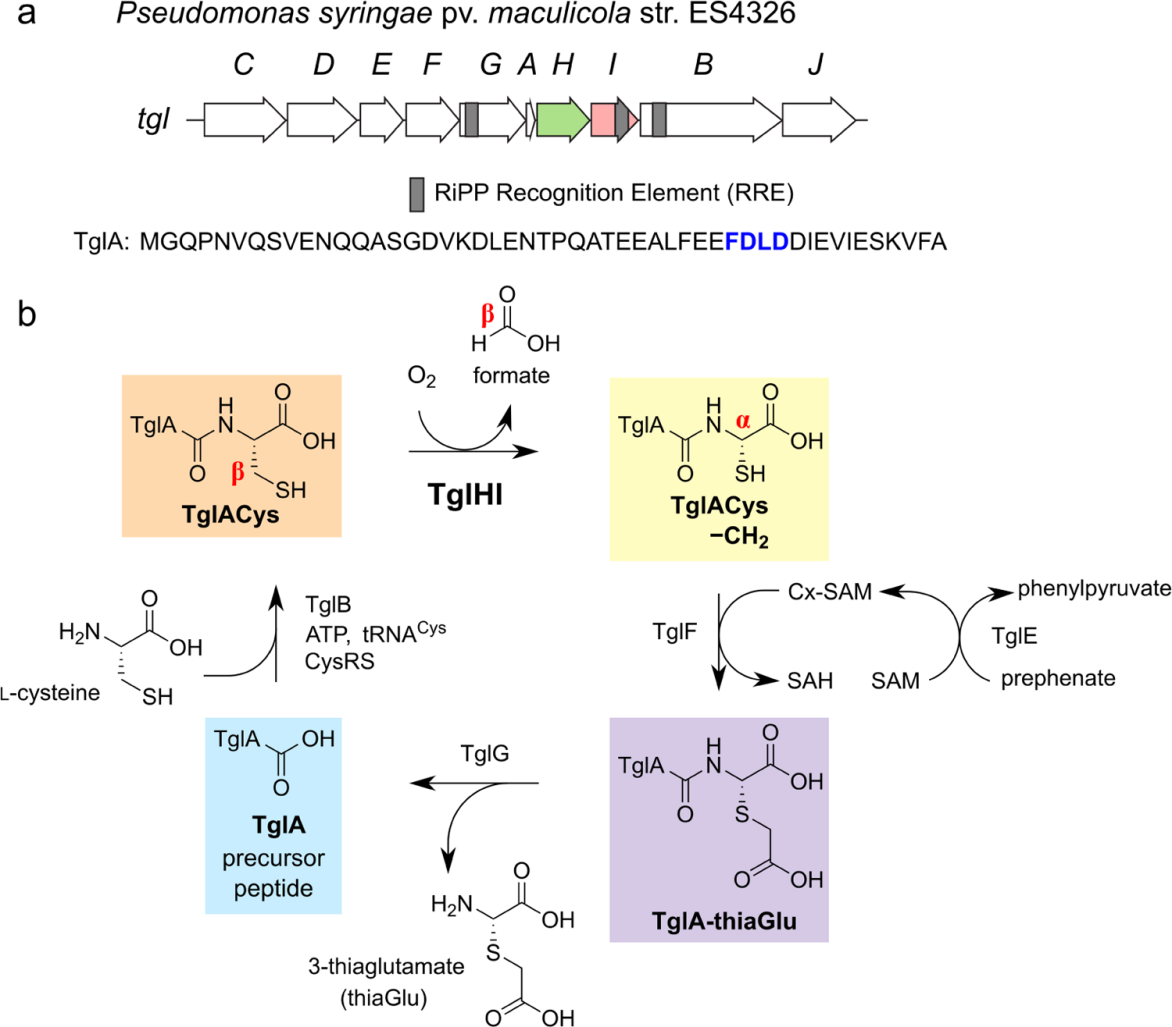
3-Thiaglutamate biosynthesis in *Pseudomonas syringae* pv. *maculicola* str. ES4326. (a) *tgl* biosynthetic gene cluster, with the *tglH* (green) and *tglI* (red) genes colored; regions encoding RiPP recognition element (RRE) domains are highlighted in gray. The F[N/D]LD LanB recognition motif of TglA is highlighted in blue. (b) Role of TglHI in the 3-thiaglutamate biosynthesis cycle. First, the ribosomally translated precursor peptide TglA (blue) is cysteinylated at its C-terminus by TglB in an ATP- and Cys-tRNA^Cys^-dependent manner to form TglACys (orange). The TglHI complex then catalyzes oxygen-dependent excision of the Cys β carbon, releasing Cβ as formate and producing an intermediate with an α-thiol group (yellow). TglE and TglF catalyze the carboxymethylation of the thiol, forming TglA-thiaGlu (purple). Finally, the membrane-associated protease TglG releases the C-terminal 3-thiaglutamate, regenerating TglA as a scaffold for the next round of biosynthesis.

The genome of the plant pathogen *Pseudomonas syringae* pv. *maculicola* str. ES4326 contains a gene cluster (*tgl*) encoding the biosynthetic machinery for the production of 3-thiaglutamate, a founding member of the recently discovered family of natural products known as pearlins (Figure 1).^1−3^ Although the physiological role of 3-thiaglutamate is still unknown, its structural similarity to glutamate suggests that it may function as an antimetabolite to interfere with glutamate signaling in plants;^1^ other pearlins are reported to have antibacterial activity (ammosamide C)^4^ and immunosuppressive properties (lymphostin).^5^ Pearlin biosynthesis has commonalities with that of ribosomally translated and posttranslationally modified peptides (RiPPs), although the pearlins themselves are not ribosomally synthesized. During pearlin and RiPP biosynthesis, a precursor peptide undergoes a series of posttranslational modifications in which the biosynthetic enzymes use a RiPP recognition element (RRE)^6^ for substrate recognition (Figure 1a). Pearlin biosynthesis is defined by a unique biosynthetic step in which a precursor peptide of ribosomal origin is nonribosomally elongated in an adenosine 5′-triphosphate (ATP)- and aminoacyl-tRNA-dependent manner by an enzyme known as a peptide aminoacyl-tRNA ligase (PEARL; Figure 1b).^3,7^ PEARLs are related to the glutamylation domains of LanB dehydratases, which catalyze the condensation of glutamate (from Glu-tRNA^Glu^) to the side chains of Ser and Thr residues in the precursor peptide during class I lanthipeptide biosynthesis.^8^ In the pearlin pathways characterized to date,^1,9^ subsequent modifications of the PEARL-appended amino acid(s) are followed by proteolysis of the nonribosomal peptide bond, yielding the original precursor peptide and a mature natural product containing no ribosomally incorporated material. The regenerated precursor may then serve as a scaffold for another round of biosynthesis (Figure 1b), a strategy reminiscent of other secondary metabolite assembly systems where intermediates are tethered to carrier proteins as thioesters (polyketide synthases, nonribosomal peptide synthetases, and the recently characterized closthioamide biosynthetic machinery)^10−13^ or amides (noncanonical amino acid biosynthetic systems in actinomycetes).^14,15^ The use of a catalytic scaffold may also save energy compared to the stoichiometric precursor peptide synthesis used for traditional RiPPs.^1^

During 3-thiaglutamate biosynthesis, TglB, the PEARL encoded in the *tgl* gene cluster, catalyzes peptide bond formation between l-cysteine (as cysteinyl-tRNA^Cys^) and the C-terminus of the 50-residue precursor peptide TglA.^1^ The scaffolded peptidyl-cysteine residue then undergoes a net four-electron oxidation consisting of excision of the Cβ methylene group, oxidation of Cβ to formate, and joining of the thiol group directly to the α position to form an (*S*)-2-mercaptoglycine residue with retention of stereochemical configuration at Cα. This complex set of reactions is catalyzed by the proteins TglH and TglI, which are insoluble separately but form a soluble TglHI complex upon coexpression of His_6_-TglH with TglI in *Escherichia coli*.^1^ TglH, the putative catalytic subunit, is a member of the DUF692 protein family (Pfam: PF05114), which also includes the nonheme iron oxidase MbnB, the catalytic subunit of the MbnBC complex that generates the oxazolone and thioamide moieties of methanobactin.^16−18^ The use of nonheme iron oxygenases and other radical-utilizing enzymes to form and break C–S bonds has been observed during the biosynthesis of a variety of secondary metabolites, such as sactipeptides and other cyclic thioether-containing RiPPs,^19,20^ ergothioneine,^21^ and quinohemoprotein amine dehydrogenase.^22^ The reactions catalyzed by TglHI and MbnBC are unique, however, and their mechanisms are incompletely understood. TglI bears no sequence homology to MbnC or other proteins of known function, but residues 171–269 are predicted by a sequence-based profile hidden Markov model to constitute an RRE.^1,6^

RRE domains are often encoded in RiPP gene clusters, where they generally mediate recognition of precursor peptides by their posttranslational modification enzymes.^6^ TglB and the peptidase TglG in the *tgl* gene cluster are also predicted to contain RREs, both of which are unrelated by sequence to the TglI RRE. TglB has been shown to bind a 20-residue C-terminal fragment of TglA as well as full-length TglA variants with single substitutions in several of the C-terminal eleven residues.^7^ Cysteinylation was observed on fragments as small as 12 residues.^1^ In contrast, preliminary activity experiments with TglHI indicated partial activity toward a 41mer TglACys C-terminal fragment lacking residues 1–10 and no activity toward further N-terminally truncated 31mer and 21mer fragments.^1^ Considered together, these results suggest that the RREs of TglB and TglI might have evolved to recognize entirely different regions of the TglA precursor peptide—a natural “hybrid” of two different RiPP posttranslational modification systems similar to other examples that are found naturally^23−28^ and to the artificial RiPP hybrids constructed recently in several laboratories.^29−34^ However, from a practical perspective, a 41mer or 51mer TglACys substrate would complicate detailed structural and mechanistic investigations of the highly unusual TglHI reaction. We therefore aimed to investigate TglHI-TglA recognition using TglACys analogs and determine the minimal region of the TglACys peptide required for TglHI activity.

## Results and Discussion

TglHI was expressed and purified as previously reported.^1^ Iron quantification using Ferene S^35^ determined that different preparations of protein contained 0.7–0.9 equiv of Fe as isolated, which increased to 2.3–2.7 equiv after reconstitution with excess Fe^2+^ and ascorbate in an anaerobic chamber. These results are consistent with the previous report of 2.5 equiv of iron after reconstitution.^1^ Both preparations catalyzed the same transformation and as-isolated TglHI was used for all *in vitro* assays as it provided the most consistent iron content.

**Figure 2:**
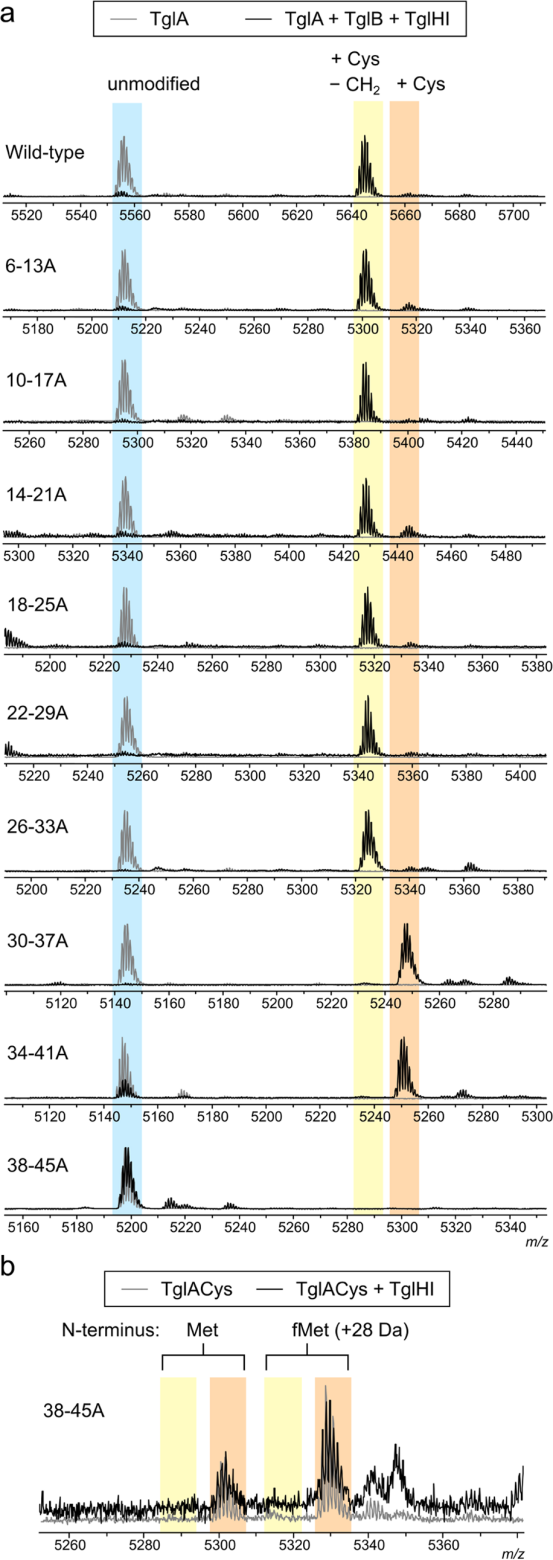
TglHI requires the C-terminal region of TglACys for activity. (a) Activity of TglB and TglHI in a coupled assay with synthetic TglA peptide variants. The assay mixture also contained Cys and *P. syringae* CysRS and tRNA^Cys^. Pairs of overlaid MALDI-TOF mass spectra depict each substrate (gray) and the product of its overnight reaction with TglB and TglHI (black). Formation of TglACys (orange) from TglA (blue) indicates TglB activity; conversion of TglACys to TglACys—CH_2_ (yellow) indicates TglHI activity. (b) Activity assay of TglHI with TglACys[38–45A] generated by IVT, colored as in (a). Translation initiation with either methionine or *N*-formylmethionine (fMet) produces two TglACys species with a mass difference of 28 Da.

We initially set out to use binding assays to identify the approximate region(s) of TglA important for TglHI recognition. However, TglA fluorescently labeled at its N-terminus did not show appreciable binding to TglHI by fluorescence polarization experiments, presumably because the label is relatively far removed from the binding site on TglA. Rather than preparing and testing a series of fluorescently labeled truncated peptides, we decided to use activity assays to determine the important regions on the substrate for catalysis. Wild-type TglA and a set of variants with overlapping (Ala)_8_ substitutions collectively spanning residues 6 through 45 (sequences in Table S1) were purchased as synthetic peptides. Each synthetic TglA peptide was incubated *in vitro* in one pot with TglB, *P. syringae* tRNA^Cys^, cysteinyl-tRNA synthetase (CysRS), and cysteine to attach the C-terminal Cys as well as with TglHI and O_2_ (in ambient air). After overnight reaction at room temperature, the products were analyzed by matrix-assisted laser desorption ionization time-of-flight (MALDI-TOF) mass spectrometry (Table S2). Consistent with previous results with truncated peptides,^7^ TglB added Cys to all TglA variants except TglA[38–45A]. TglHI catalyzed the oxidation of the C-terminal cysteine added to the wild-type TglA and (Ala)_8_ variants TglA[6–13A] through TglA[26–33A] but not TglA[30–37A] or TglA[34–41A] (Figure 2a). Because TglB did not cysteinylate TglA[38–45A], TglHI activity could not be assessed for this peptide; instead, TglACys[38–45A] was synthesized on a small scale from a double-stranded DNA (dsDNA) template by *in vitro* transcription-coupled translation (IVT)^36^ using a commercially available kit^37^ (see the Methods section) and then reacted with TglHI for 2 h. No turnover was visible by MALDI-TOF MS (Figure 2b).

**Figure 3:**
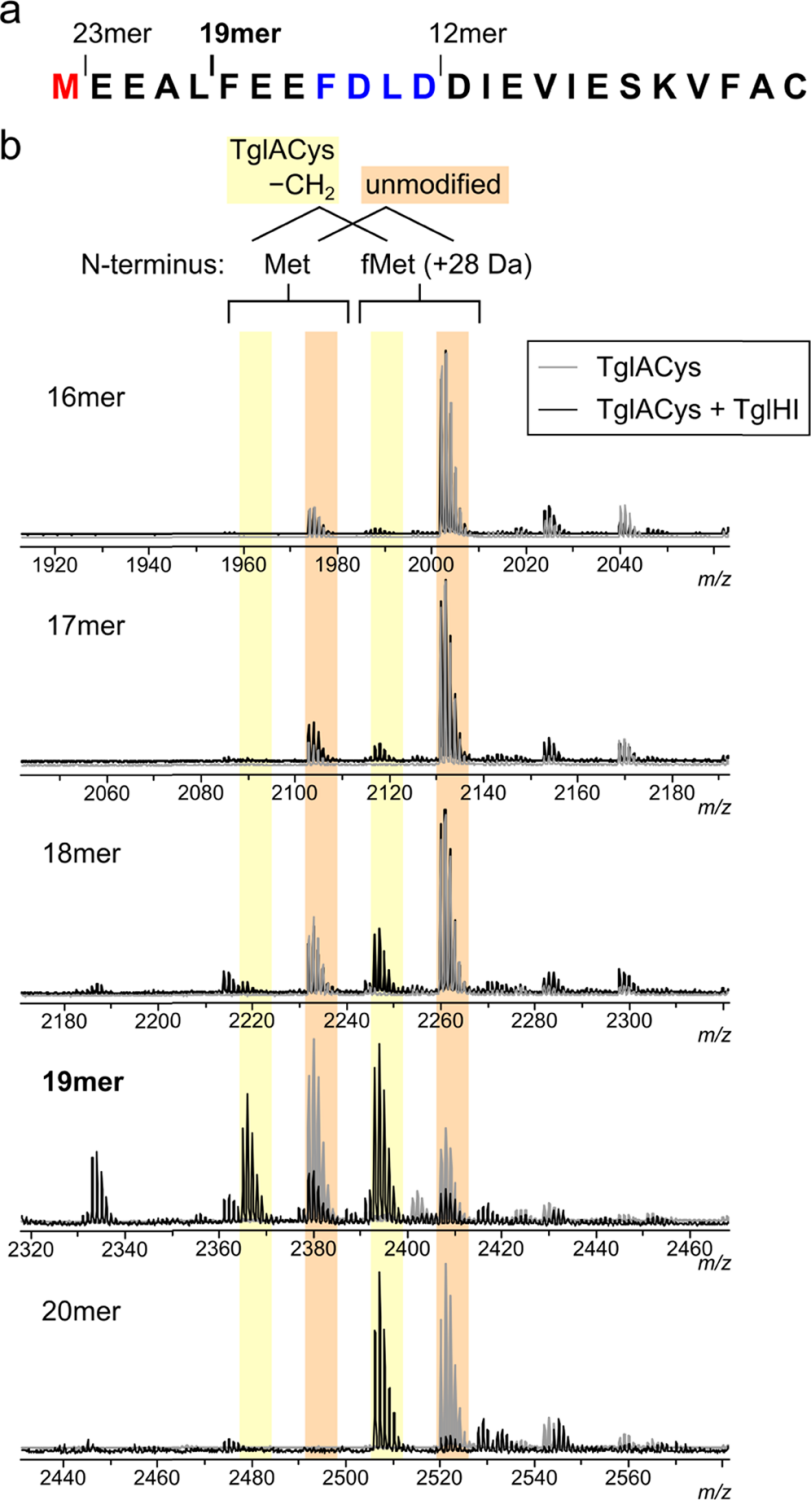
TglHI activity toward C-terminal fragments of TglACys generated by IVT. (a) Sequence of the longest fragment tested (23mer) with the F[N/D]LD motif in blue and the N-terminal Met introduced during translation initiation in red. Start sites (excluding the required N-terminal Met for each peptide) of the longest and shortest fragments tested are marked with lines; the 19mer was used for subsequent mutagenesis. (b) Pairs of MALDI-TOF mass spectra depicting IVT-generated C-terminal fragments of TglACys (gray) and the products of 2 h *in vitro* reactions with TglHI (black). See Figure S1 for MS data with peptides shorter than the 16mer and longer than the 20mer.

The observed activity of TglHI with TglACys[26–33A] but not with TglACys[30–37A], TglACys[34–41A], or TglACys[38–45A] suggested that residues C-terminal to Glu34 of TglACys might be necessary for TglHI recognition. To determine if these residues are also sufficient for catalysis, short DNA templates encoding an initiator methionine followed by C-terminal fragments of TglACys beginning with residues Glu29 (23mer) through Asp40 (12mer) were constructed from synthetic oligonucleotides and the corresponding peptides were prepared by IVT (Figure S1; see Table S3 for sequences). TglHI activity was detectable *in vitro* toward the 17mer and increased with peptide length, nearly depleting the 20mer substrate in 2 h (Figure 3). The 19mer fragment (20 residues total including the N-terminal Met) was then used as a starting point for subsequent mutagenesis experiments. This peptide is shorter than the heterologously expressed 31mer that in a previous study was not converted by TglHI.^1^ The previous study was conducted at 100 μM peptide, whereas IVT results in much lower concentrations. It is possible that peptide aggregation may have led to the previous conclusion that TglHI requires a longer peptide.

**Figure 4:**
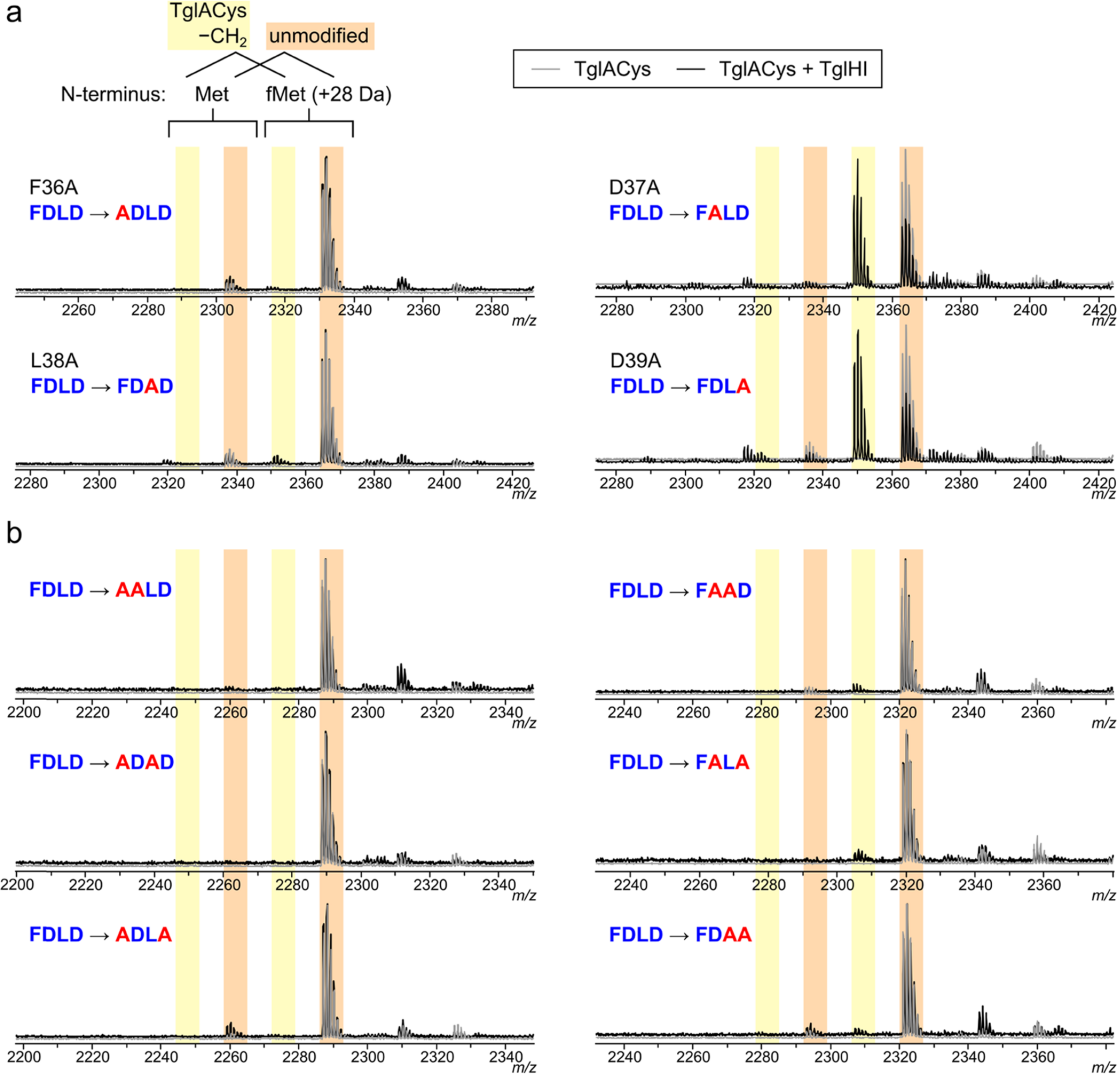
TglHI activity toward IVT-generated variants of the TglACys 19mer with alanine substitutions in the F[N/D]LD motif. Pairs of overlaid MALDI-TOF MS spectra depict peptide variants (gray) and the products of 2 h *in vitro* reactions with TglHI (black). (a) Single mutations in the hydrophobic residues (left) and acidic residues (right). (b) Double mutations with residue F36 mutated (left) or retained (right).

Considering the importance of the F[N/D]LD sequence motif for LanB binding to class I lanthipeptide precursors^8,38,39^ and the presence of this motif in the TglA sequence (Figure 1a), single and double alanine substitutions in Phe36–Asp39 of TglACys were generated by IVT and investigated for their effects on TglHI activity *in vitro*. Turnover was abolished on the F36A variant and barely detectable for L38A, but the D37A and D39A variants were converted at comparable efficiency to the original 19mer (Figure 4a). Of the six possible double variants, TglHI did not act on the three containing the F36A mutation; activity was barely detectable toward D37A/L38A, L38A/D39A, and D37A/D39A (Figure 4b). Taken together, these results indicate that Phe36 is required and Leu38 is important for TglACys recognition by TglHI, explaining the lack of TglHI activity toward the full-length 30–37A and 34–41A peptides (Figure 2). The presence of either Asp37 or Asp39 is also important, but substitution of one residue is moderately tolerated if the other is present. These findings reveal the similarity between the peptide recognition modes of TglHI and class I lanthipeptide synthetases (LanBs) such as NisB, which recognize the F[N/D]LD motifs of LanA precursor peptides primarily through hydrophobic interactions, especially with the Phe and Leu residues of the motif.^8,40−42^ Notably, this recognition mode is not shared by TglB, which is closely related to LanBs, but accepts a 12mer TglA substrate that only contains the final Asp residue of the motif.^1^

**Figure 5:**
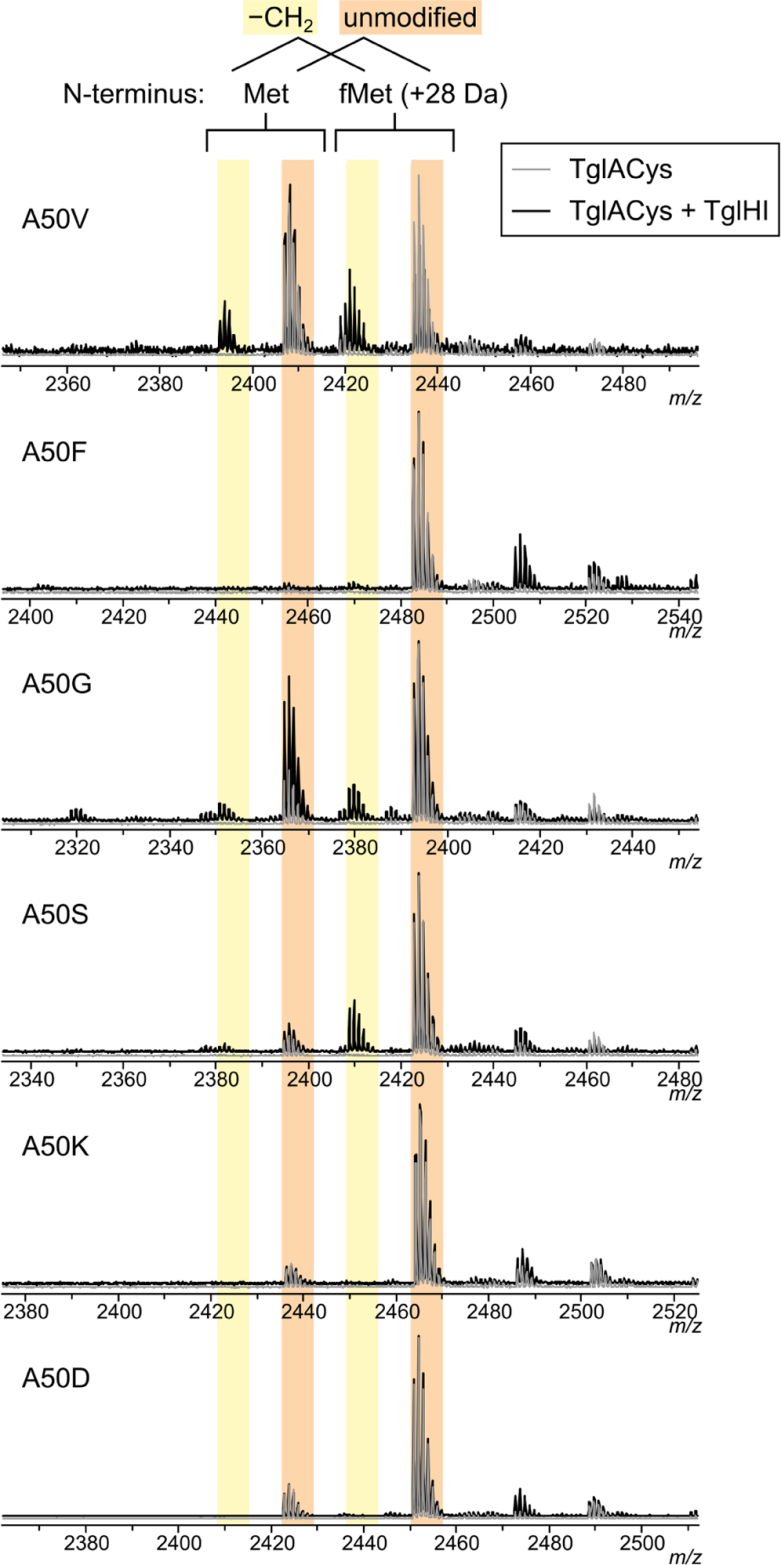
TglHI specificity with respect to the penultimate residue (Ala50) of TglACys. Pairs of overlaid MALDI-TOF mass spectra depict IVT-generated TglACys 19mer peptide variants (gray) and the products of 2 h *in vitro* reactions with TglHI (black).

Additional recognition determinants in the TglACys sequence were identified in the same manner through a systematic alanine replacement scan of the 19mer as well as insertions and deletions in residues 46–50. No single-alanine substitution outside the F[N/D]LD motif completely abolished modification by TglHI, but only the E42A, V43A, E45A, and S46A variants were well tolerated; mutations in the remaining residues caused a significant reduction in activity (Table 1 and Figure S2). Alanine insertions at positions 46 and 47 resulted in partial turnover, whereas insertions at positions 49 and 50 abolished modification and only slight modification was observed with the Ins48A mutant; deletions at any of the five C-terminal positions were not tolerated (Table 1 and Figure S3). The identity of Ala50, the residue flanking the Cys that TglHI modifies, was also important for recognition, with replacements with Ser, Gly, or Val leading to low levels of modification and substitutions with Phe, Lys, or Asp preventing modification entirely (Figure 5). Thus, it appears that the recognition of TglACys by TglHI involves both the Phe and Leu of the F[N/D]LD motif remote from the site of modification and additional residues nearer the Cys that is modified.

**Table 1:** Modification of TglACys 19mer Point Mutants by TglHI^a^

variant	activity^b^	variant	activity^b^
F49A	+	Ins50A	–
V48A	+	Ins49A	–
K47A	+	Ins48A	+
S46A	+++	Ins47A	++
E45A	+++	Ins46A	++
I44A	+	ΔA50	–
V43A	+++	ΔF49	–
E42A	+++	ΔV48	–
I41A	+	ΔK47	–
D40A	+	ΔS46	–
E35A	+		
E34A	++	wild-type	+++
F33A	+		

a For MS data, see Figure S2.

b Activity values reflect estimated substrate conversion based on the intensity of MALDI-TOF MS signals: −, no product visible; +, <33% conversion; ++, 33–67% conversion; +++, >67% conversion.

**Figure 6:**
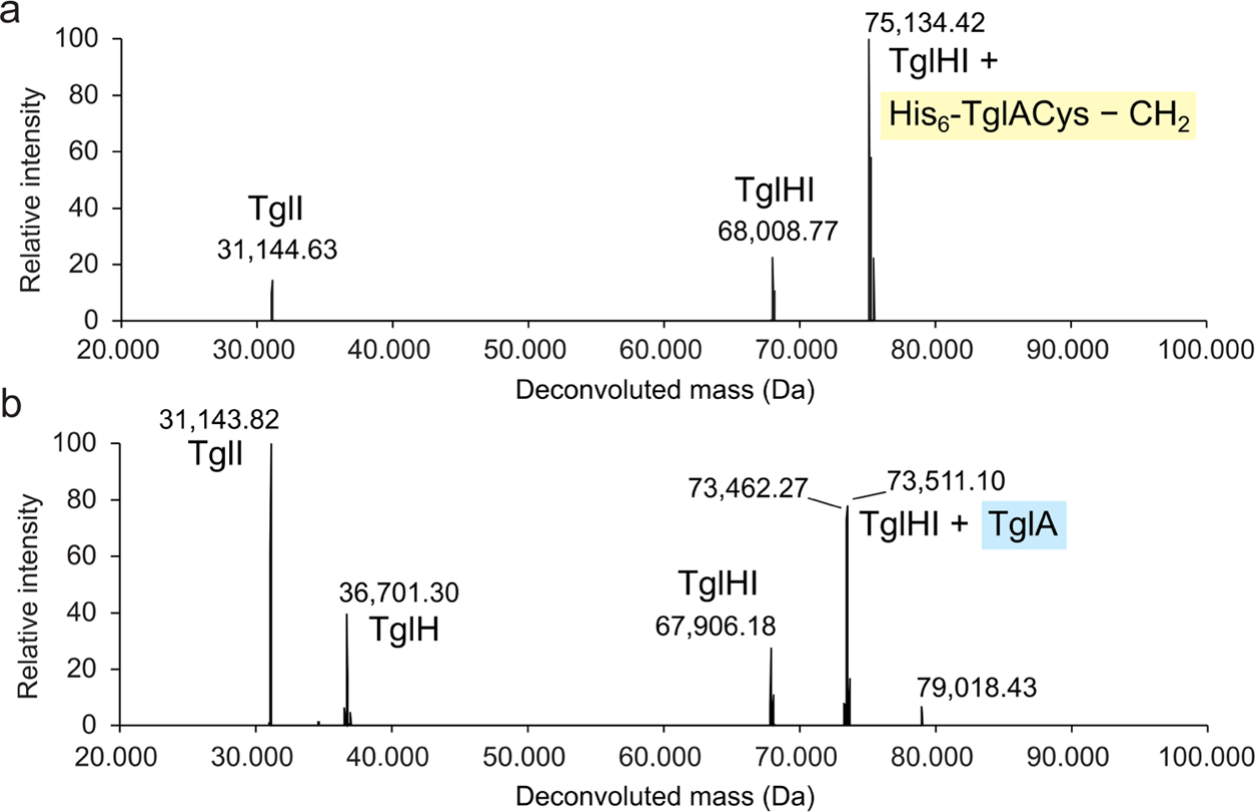
Binding of His_6_-TglA-Cys—CH_2_ or TglA to purified TglHI shown by nMS. (a) Binding of TglHI to the product of its reaction with His_6_-TglACys. (b) Binding of TglHI to TglA. The calculated average mass of His_6_-apo-TglH is 36,702.4 Da, and the observed mass after deconvolution is 36,701.3 Da. The calculated average mass of TglI is 31,014.3 Da, and the observed mass after deconvolution is 31,143.4 Da. Higher masses of proteins than their calculated molecular weights have been observed previously in nMS and have been attributed to incomplete desolvation,^51^ but we cannot rule out a covalent modification during expression. The formation of a TglHI heterodimer and TglHI bound to the substrate suggests that the proteins maintain a folded conformation under the conditions employed.

We also investigated whether the binding site of TglACys is predominantly in the RRE-containing TglI protein, or the active site of the TglH protein, or both. Because TglH and TglI could not be expressed individually and could not be separated after expression and purification, native mass spectrometry (nMS) was used to observe the intact TglHI complex and partially dissociate the subunits. Analysis of TglHI by nMS in the presence of the substrate TglACys led to the detection of the TglHI complex bound to what appears to be the product of the reaction (Figure 6a) as the increase in mass compared to the apo complex was 7125.65 Da (average mass of N-terminally His_6_-tagged TglACys—CH_2_ = 7126.58 Da). When the TglHI complex was incubated with TglA (*i.e*., lacking the C-terminal Cys), the analogous complex was observed (Figure 6b). Hence, the Cys does not appear to be required for binding to the TglHI heterodimer. The monomers corresponding to TglH and TglI were also observed in the spectrum, but they were not bound to the scaffold peptide. Therefore, it appears that peptide binding to TglHI involves extensive interactions with both TglH and TglI even in the absence of Cys51.

To provide a visual approximation of the TglHI complex and its interaction with the substrate, the AlphaFold-Multimer algorithm^43^ was used to predict the structure of a 1:1 heterodimer of TglHI both with and without the TglACys 19mer (Figure S4a). In the models, TglH did not contain iron, but in the trimeric complex, the C-terminal Cys of the 19mer was still located in the vicinity of the iron ligands (based on the structure of another DUF692 family member, PDB 3BWW, and a recent study on the DUF692 enzyme MbnB^18^). The predicted structure of apo-TglH in the complex with TglI is similar to that of MbnB, illustrating the capability of AlphaFold (the prediction was performed before the MbnB structure was reported). MbnB contains three irons in the crystal structure, and the MbnABC complex shows threading of the substrate MbnA from MbnC to the active site of MbnB where a cysteine in the core peptide makes a direct contact with one of the irons.^18^ In the predicted TglHI complex with the 19mer peptide of TglA, a very similar interaction is seen that starts at TglI and ends in the active site of TglH. In the AlphaFold model, the C-terminal domain of TglI adopts an RRE fold (three antiparallel β-strands followed by three α-helices), and the substrate TglA binds to the RRE; MbnC does not contain a canonical RRE fold, but its C-terminal domain contains a β-sheet that makes an antiparallel β-sheet interaction with the MbnA leader peptide^18^ similar to the way substrate binds to β3 in RREs in other structurally characterized RiPP systems.^3,44^ Thus, the predicted model of the 19mer binding to TglHI recaptures several of the features seen in the crystal structure of the related MbnABC protein–substrate complex.

The TglHI model (with or without the substrate) predicts a distance of 50–55 Å from the putative metal-binding site in TglH to the predicted “hydrophobic cage” formed between β3, α1, and α3 of the RRE that interacts with the Phe residue of the F[N/D]LD motif in the NisB/NisA system (Figure S4b).^8^ If these features function similarly in TglHI, the sequence of TglACys that should span this distance, Phe36 to Cys51, would have a maximum extended length of about 56–64 Å (based on contour lengths of 3.5–4.0 Å per residue).^45^ Therefore, the predicted structure of the TglHI heterodimer is consistent with a binding mode in which (1) the F[N/D]LD motif of TglACys binds to the RRE of TglI; (2) the C-terminal cysteine binds to the iron center of TglH; and (3) intervening residues such as Asp40, Ile41, and Ile44 make additional key contacts across the TglH–TglI interface, as indicated by sluggish TglHI-catalyzed modification of alanine variants at these positions (Table 1 and Figure S2).

However, the binding pose of TglACys interacting with TglHI predicted by AlphaFold-Multimer deviates significantly from the anticipated interaction. Although the predicted structure shows the TglACys leader peptide making antiparallel β strand interactions with β1 of the TglI RRE as observed in other systems, Phe36 of the F[N/D]LD motif does not interact with the hydrophobic cage, in contrast to the NisA–NisB complex. Instead, Phe36 interacts with an extended loop between β1 and β2 of the RRE that is closer to the TglH active site, and Phe33 of TglACys interacts with the hydrophobic cage (Figure S4d,e). The length of this loop is unique to TglI among structurally characterized RREs^8,44,46−50^ (Figure S4c). The predicted binding model does not explain several of our experimental observations. First, Phe33 is less critical than Phe36 for modification of TglACys by TglHI (Table 1 and Figure 4). Second, the interaction of Phe36 with the loop in TglI rather than the hydrophobic cage means that the distance between Phe36 and the active site would be only 35–40 Å (Figure S4b). If this prediction is correct, then the observation that TglHI did not process the deletion mutants cannot be due to the inability of the C-terminal Cys to reach the active site and instead must reflect specific interactions in the substrate tunnel that cannot be made in these variants. Future structural biology studies will be needed to resolve these questions.

**Figure 7:**
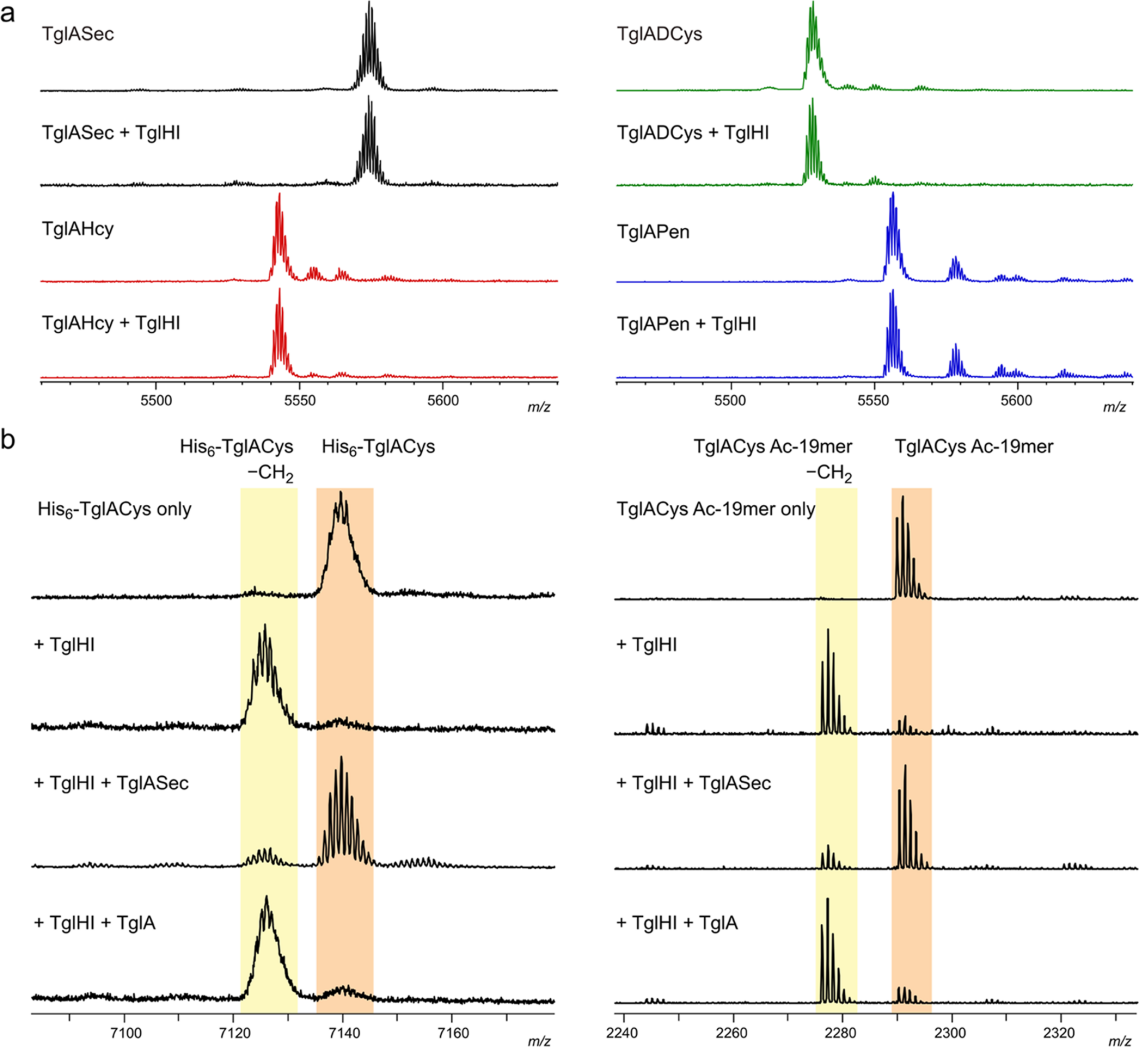
TglACys analogs with noncanonical C-terminal residues. (a) MALDI-TOF mass spectra of *in vitro* reaction mixtures containing 100 μM TglASec (black), TglAHcy (red), TglADCys (green), or TglAPen (blue) after 4 h at room temperature with or without 10 μM TglHI. The TglASec sample also contained TglA as a major impurity, which is not shown in the spectral window (see also Figure S5a). (b) Inhibition of TglHI by TglASec. MALDI-TOF MS spectra of 50 μM test substrate (top traces; His_6_-TglACys at left, TglACys Ac-19mer at right) and room-temperature reactions containing 50 μM substrate, 5 μM TglHI, and either no inhibitor, 50 μM TglASec, or 50 μM TglA are shown. TglHI-catalyzed modification of the substrate is nearly complete after 30 min; however, the addition of TglASec prevents any appreciable turnover during the same time. TglA (lacking Cys) at the same concentration does not inhibit turnover (bottom traces).

To further characterize the role of Cys51 in TglACys recognition by TglHI, a set of TglACys analogs with noncanonical amino acid residues replacing Cys51 was generated by expressed protein ligation (EPL).^52,53^ TglA was expressed as a C-terminal fusion with a temperature-dependent intein and chitin-binding domain (CBD),^7^ intein catalysis was induced to generate a TglA C-terminal thioester *in situ*, and the thioester was cleaved with either l-selenocysteine (Sec), l-homocysteine (Hcy), d-cysteine, or l-penicillamine (β-dimethyl-l-cysteine, Pen) to generate the corresponding peptide bond.^53^ The resulting peptides (TglASec, TglAHcy, TglADCys, and TglAPen, respectively) were purified and used for *in vitro* TglHI activity assays. TglASec initially contained a peptide impurity of similar mass that could not be resolved by high-performance liquid chromatography (HPLC) but was readily hydrolyzed to TglA under basic conditions (Figure S5a); after hydrolysis, the resulting mixture of TglASec and TglA was used without further purification. TglHI did not excise the β-carbon atom from any of the four unnatural analogs (Figure 7a); however, the TglASec sample inhibited modification of both full-length His_6_-TglACys and a synthetic N-terminally acetylated 19mer (sequence Ac-FEEFDLDDIEVIESKVFAC, “TglACys Ac-19mer”) when the TglACys substrate and TglASec were present at equal concentrations (Figure 7b). None of the other three non-natural TglACys analogs were inhibitory under the same conditions (Figure S5b). Wild-type TglA did not inhibit TglHI, indicating that the inhibitory species in the TglASec sample is TglASec rather than TglA (Figure 7b). The mechanism of TglHI inhibition by TglASec is under further investigation.

## Conclusions

The experiments described herein used IVT for rapid high-throughput generation and characterization of variant TglACys peptide sequences, allowing the systematic identification of substrate residues important for *in vitro* recognition and β-carbon excision by TglHI. The results of the coupled TglB-TglHI activity assays corroborate previous observations that TglB-catalyzed cysteinylation of the TglA C-terminus does not require the N-terminal 38 residues of TglA.^1^ In contrast, TglHI-catalyzed Cβ excision from TglACys is strongly dependent on the F[N/D]LD motif comprising residues Phe36–Asp39, which has been shown in class I lanthipeptide systems to mediate substrate recognition by LanBs.^8^ TglB and other PEARL enzymes share a common ancestor with LanBs, whereas the TglI sequence (including its RRE) is not homologous to any protein with known function. The identity of the C-terminal Cys residue is also critical for catalysis by TglHI; the analogs TglA (lacking the Cys), TglAHcy, TglADCys, and TglAPen are not modified by TglHI *in vitro* and do not inhibit TglHI-catalyzed modification of TglACys peptides in competition experiments. However, the substrate analog TglASec nearly completely inhibits TglHI at equimolar concentrations with the substrate, though TglASec itself does not undergo Cβ excision.

This study also demonstrates that a synthetic 19mer peptide corresponding to the C-terminus of TglACys is a minimal substrate for TglHI to catalyze its carbon excision reaction. This peptide is significantly shorter than the previously reported length requirement and shows that both TglB and TglHI can act on peptides that consist of the C-terminus of the TglA sequence. For TglB, the minimal requirement is the final 12 amino acids, whereas TglHI requires the final 19 amino acids for catalysis, indicating overlapping but not identical substrate binding requirements. Structure prediction by AlphaFold-Multimer appears to predict the structures of TglHI well based on a recent crystal structure of MbnBC,^18^ but the predicted mechanism of substrate engagement either suggests a new type of interaction between the RRE and substrate or illustrates shortcomings of the current capabilities for predicting interactions of short peptides with their modifying enzymes.

*In vitro* assays with single-alanine variants of the 19mer showed that the F[N/D]LD motif comprising TglA residues 36–39 is critical for TglHI catalysis, as well as residues Asp40, Ile41, and Ile44 between the motif and the C-terminal Cys residue. Studies with insertion and deletion mutants suggest that TglHI also requires a specific distance from the putative binding site on the substrate to the C-terminal Cys for the carbon excision reaction to occur efficiently. These studies reported on activity and hence we cannot distinguish whether the peptide variants that were not accepted do not bind to the enzyme or bind in a nonproductive fashion. The discovery of a short, synthetically accessible substrate and a potent inhibitor for TglHI should serve as a springboard for more detailed mechanistic and structural characterization of this remarkable reaction.

## Methods

For materials, expression and purification of enzymes and substrates, and MS procedures, see the Supporting Information.

### TglB-Coupled TglHI Activity Assays with Synthetic Substrates

TglA (10 μM) was mixed with 5 μM TglHI, 0.5 μM His_6_-TglB, 50 mM 4-(2-hydroxyethyl)piperazine-1-ethanesulfonic acid (HEPES) pH 7.5, 100 mM NaCl, 10 mM MgCl_2_, 5 mM ATP, 2 mM l-cysteine HCl, 1 mM TCEP–HCl, 2.5 μM *P. syringae* tRNA^Cys^, and 0.5 μM *P. syringae* CysRS in a total volume of 100 μL. Reactions were initiated with His_6_-TglB and incubated overnight at 30 °C. After 18 h, reaction mixtures were acidified by addition of trifluoroacetic acid (TFA) to a final concentration of 0.3% (v/v), desalted using C18 ZipTips, and analyzed by MALDI-TOF MS.

### Transcription-Coupled *In Vitro* Translation (IVT) and TglHI Activity Assays with IVT-Generated Peptides

DNA templates for IVT (except for TglACys[38–45A], see the Supporting Information) were generated from single-stranded oligonucleotides (Table S4) by two rounds of 16-cycle overlap extension PCR (one round for the TglACys 12mer template) with Taq polymerase. Each first-round PCR mixture served as the template for the second round of PCR by direct 1:100 dilution into the second-round reaction. Primers and annealing temperatures for each template are listed in Table S5. Amplified templates were precipitated with ethanol and redissolved in a minimal volume of RNase-free ddH_2_O for IVT.

In a typical IVT reaction, dsDNA template (3 μL, approx. 6 μg) was mixed with NEB PURExpress *in vitro* Protein Synthesis Kit reagents A (4 μL) and B (3 μL) on ice in a prelubricated RNase-free 0.6 mL microcentrifuge tube. The reaction was incubated at 37 °C for 1 h on a prewarmed heat block and divided into 2.5 μL aliquots. One aliquot was immediately desalted and analyzed by MALDI-TOF MS; each remaining aliquot was used in a 10 μL activity assay containing 5 μM TglHI in 0.7× reaction buffer (1×: 50 mM Na_2_HPO_4_, 300 mM NaCl, 10% [v/v] glycerol, pH 7.6). Assay mixtures were incubated at room temperature in ambient air for 2 h, desalted, and analyzed by MALDI-TOF MS.

### TglHI Activity Assays with Purified Substrates

In a typical assay, 10 μM TglHI was added to 100 μM of substrate peptide in 1× reaction buffer in a final volume of 100 μL. Inhibition assays contained 50 μM of inhibitor peptide, 50 μM of substrate peptide, and 5 μM TglHI. After initiation with TglHI, reactions were incubated at room temperature (23 °C); at various time points, 20 μL aliquots were withdrawn, desalted, and analyzed by MALDI-TOF MS.

## Supporting Information Available

Sequence of the codon-optimized TglACys[38–45A] gene, materials used, detailed procedures for protein expression, purification, and activity assays, Figures S1–S7, and Tables S1–S5 (PDF)

## Accession Codes

NCBI Protein accessions: TglH, WP_007252215.1; TglI, WP_007252216.1; TglA, WP_007252214.1; TglB, WP_007252217.1. Nucleotide accessions: *P. syringae* pv. *maculicola* str. ES4326 genome, NZ_CP047260.1.

## Abbreviations

4-MPAA: 2-(4-mercaptophenyl)acetic acid
ATP: adenosine 5′-triphosphate
CBD: chitin-binding domain
DTT: dl-dithiothreitol
dsDNA: double-stranded DNA
DTNB: 5,5′-dithiobis(2-nitrobenzoic acid)
EDTA: ethylenediaminetetraacetic acid
EPL: expressed protein ligation
ESI: electrospray ionization
fMet: l-(*N*-formyl)methionine
HEPES: 4-(2-hydroxyethyl)piperazine-1-ethanesulfonic acid
HPLC: high-performance liquid chromatography
IMAC: immobilized metal affinity chromatography
IVT: *in vitro* transcription-coupled translation
LB: Luria-Bertani medium
MALDI: matrix-assisted laser desorption ionization
MWCO: molecular weight cutoff
nMS: native mass spectrometry
PEARL: peptide aminoacyl-tRNA ligase
PMSF: phenylmethanesulfonyl fluoride
RiPP: ribosomally translated and posttranslationally modified peptide
RRE: RiPP recognition element
SAH: *S*-adenosyl-l-homocysteine
SAM: *S*-adenosyl-l-methionine
SPE: solid-phase extraction
ssDNA: single-stranded DNA
TB: Terrific Broth
TCEP: tris(2-carboxyethyl)phosphine
TFA: trifluoroacetic acid
TOF: time of flight
Tris: tris(2-hydroxymethyl)aminomethane
tRNA^Cys^: cysteine transfer RNA
