# Supplementary material for: Substrate Recognition by the Peptidyl-(S)-2-mercaptoglycine Synthase TglHI during 3-Thiaglutamate Biosynthesis
Source: ACS Chem Biol. 2022 Apr 1;17(4):930–40. doi: 10.1021/acschembio.2c00087 (PMC9016710; doi:10.1021/acschembio.2c00087)
Supplement: Supplementary file 1 — cb2c00087_si_001.pdf [file cb2c00087_si_001.pdf]

Supporting Information

Substrate recognition by the peptidyl-(*S*)-2-mercaptoglycine synthase TglHI during 3-thiaglutamate biosynthesis

Martin I. McLaughlin,[a‡] Yue Yu,[a] and Wilfred A. van der Donk[*ab]*

[a]Department of Chemistry and Carl R. Woese Institute for Genomic Biology, University of Illinois at Urbana-Champaign, Urbana, Illinois 61801, United States
[b]Howard Hughes Medical Institute, University of Illinois at Urbana-Champaign, Urbana, Illinois 61801, United States
‡ Current address: Department of Bioengineering, Stanford University, Stanford, CA 94305, USA.

[*] Corresponding author: vddonk@illinois.edu

Table of Contents

SI Materials and Methods S2

Sequence of codon-optimized synthetic gene for TglACys[38-45A] S5

Table S1. Sequences of synthetic TglA peptides S6

Table S2. Observed and theoretical *m/z* ratios for peptide ions in TglB-TglHI coupled assays...... S6

Table S3. Sequences of short TglACys peptides generated by IVT S7

Table S4. Oligonucleotide sequences S8

Table S5. PCR conditions for construction of TglACys fragment IVT templates S10

Figure S1. TglHI activity toward longer and shorter TglACys fragments S12

Figure S2. TglHI activity toward 19mer TglACys peptides with alanine substitutions........ S13

Figure S3. TglHI activity toward 19mer TglACys peptides with insertions and deletions........ S14

Figure S4. Predicted structure of the TglHI complex with the TglACys 19mer using AlphaFold-Multimer S15

Figure S5. Unnatural analogues of TglACys S17

Figure S6. TglHI-peptide complex native MS spectra before deconvolution................................ S18

Figure S7. ESI-MS analysis of TglASec after GluC digestion and iodoacetamide alkylation....... S19

References.. S20

SI Materials and Methods

Materials. His_6-TglB and *P. syringae* His_6-CysRS were expressed and purified as described previously.[1] *P. syringae* $tRNA^{Cys}$ was generated by *in vitro* transcription as described previously.[2] Synthetic TglACys Ac-19mer, TglA and variant peptides were purchased from GenScript; all peptides exceeded 95% purity except TglA[14-21A] (94.9%). Oligonucleotides for construction and amplification of IVT templates were obtained from Integrated DNA Technologies (Coralville, IA); sequences are listed in Table S4. A codon-optimized synthetic gene for IVT of TglACys[38-45A] was obtained from Twist Biosciences (South San Francisco, CA) and amplified by PCR; the sequence is listed on page S5. Chitin resin and the PURExpress® *In Vitro* Protein Synthesis Kit used for IVT were from New England Biolabs (Ipswich, MA). Ammonium acetate, ascorbic acid, chloramphenicol, L-cysteine, 5,5′-dithio-bis(2-nitrobenzoic acid), Ferene S, formate dehydrogenase (from *Candida boidinii*), L-selenocystine, and Super DHB were purchased from Sigma (St. Louis, MO); L-homocystine, D-cysteine, L-penicillamine, and 4-mercaptophenylacetic acid (4-MPAA) were from Chem Impex (Wood Dale, IL). HisPur Ni-NTA Superflow resin and Zeba spin desalting columns were from Thermo Fisher Scientific (Waltham, MA). Ampicillin sodium salt, DL-dithiothreitol (DTT), isopropyl β-D-1-thiogalactopyranoside (IPTG), kanamycin monosulfate, lysozyme, tris(2-carboxyethyl)phosphine hydrochloride (TCEP-HCl), and Tris were from GoldBio (St. Louis, MO). HEPES was from Calbiochem (San Diego, CA). Trifluoroacetic acid (TFA) was from Acros Organics (Geel, Belgium) and Sigma; acetonitrile (MeCN) was from Fisher Scientific (Waltham, MA). Iron standard solutions were from SPEX CertiPrep (Metuchen, NJ) or Sigma (St. Louis, MO). ZipTip 0.6-µL C18 desalting pipet tips were from Millipore (Burlington, MA). All other chemicals were of reagent grade or higher.

Expression and purification of TglHI. His_6-TglHI was expressed and purified as previously described[1] with modifications as follows: Rosetta 2 (DE3) cells were used for expression and 34 µg/mL chloramphenicol was added to all cultures; expression was induced with 1 mM IPTG for 12-18 h; Tris buffer in lysis, wash, and elution buffers was replaced with 20 mM HEPES, pH 7.5; cells were lysed by sonication; resin volume was decreased to 2.5 mL/L expression culture; column wash was decreased to 2 × 5 column volumes. Final storage buffer contained 20 mM Tris (pH 7.6 @ 4 °C), 300 mM NaCl, and 15% [v/v] glycerol. Protein concentration was estimated by absorbance at 280 nm using an extinction coefficient of 112,760 M^{-1} cm^{-1} calculated by ExPASy (expasy.org/protparam) for a 1:1 complex of His_6-TglH and TglI; iron content was analyzed by the method of Beinert.[3] Addition of 1 mM $Fe(NH_4)_2(SO_4)_2$ to the growth medium followed by protein purification in a Coy anaerobic chamber did not improve TglHI yield or iron content.

Iron reconstitution of TglHI. Reconstitution of His_6-TglHI was performed in a Coy vinyl anaerobic chamber with an atmosphere of 96-97% N_2/3-4% H_2 and O_2 level kept below 10 ppm. Concentrated solutions of $FeSO_4 \cdot 7H_2O$ (100 mM) and ascorbic acid (1 M, pH 7 with NaOH) were diluted in storage buffer to create an ice-cold stock solution of 10 mM $FeSO_4$ + 20 mM ascorbate. His_6-TglHI was mixed with the stock solution in a molar ratio of 3.3 Fe/TglHI. After 1 h incubation on ice, TglHI was desalted using a PD-10 column (Cytiva, Marlborough, MA) equilibrated in ice-cold oxygen-free storage buffer, concentrated in the anaerobic chamber, flash frozen, and stored at −80 °C. Protein concentration and iron content was determined as above.

Expression and purification of His_6-TglACys. His_6-TglACys was expressed and purified as previously described[1] with modifications as follows: Rosetta 2 (DE3) cells were used for expression and 34 µg/mL chloramphenicol was added to all cultures; cultures were grown for 8 h before harvest; 1 mg/mL lysozyme, 100 U/mL DNase I, 1 mM TCEP, and protease inhibitor (Roche cOmplete) were added to the lysis buffer; IMAC was performed by gravity flow using 10 mL of Ni-NTA resin per 4 L culture; two wash steps were performed with 5% and 10% elution buffer, respectively; after buffer exchange into ddH_2O, 1 mM TCEP was added and the peptide was lyophilized, redissolved in 0.2% (v/v) TFA, filtered, and purified directly by HPLC without an intermediate solid-phase extraction step. HPLC was performed using an Agilent 1200 instrument and a Waters XBridge C18 Prep column (10 × 250 mm, 5 µm) equilibrated in 95% solvent A (ddH_2O + 0.1% TFA)/5% solvent B (MeCN + 0.1% TFA) at a flow rate of 5 mL/min. After sample injection, a 2-min isocratic hold of 5% B was followed by a 20-min linear gradient of 5-100% B. His_6-TglACys eluted at approx. 12.1 min under these conditions. Lyophilized peptide was redissolved in ddH_2O, adjusted to pH 6-7 with NH_4OH, and stored at −80 °C.

Expression and purification of TglA-intein-CBD fusion protein. *E. coli* BL21(DE3) cells were transformed with pTXB1:TglA plasmid;[2] 100 mL of overnight culture was harvested, resuspended in 4 × 20 mL of fresh LB + 100 µg/mL ampicillin (Amp), and inoculated into 4 × 2 L of pre-warmed TB + Amp. Cultures were shaken at 37 °C and 200 rpm to an OD_{600} of 0.6 and cooled on ice before induction with 0.4 mM IPTG. After 14 h overexpression at 16 °C and 200 rpm, cells (115 g) were harvested at 8,000 × g and 0 °C and resuspended in an anaerobic chamber (see "Iron reconstitution of TglHI" above) in 300 mL of fusion buffer (20 mM HEPES pH 7.5, 500 mM NaCl, 1 mM EDTA) supplemented with 2 mM PMSF, 1 µM leupeptin, and 1 µM protease inhibitor E-64. All purification buffers were oxygen-free and kept ice-cold. Cells were lysed using an Avestin Emulsiflex C3 homogenizer (Ottawa, Canada) for 2 cycles at ~ 10,000 psi and the lysate was centrifuged at 17,700 × g and 0 °C for 1 h. Supernatant (approx. 440 mL) was kept on ice in the anaerobic chamber and loaded (1 mL/min) onto 4 × 30 mL chitin resin columns equilibrated in fusion buffer. Each column was washed (1-2 mL/min) with 225 mL of fusion buffer containing 0.05% [v/v] Triton X-100 followed by 100 mL of fusion buffer. Intein cleavage was induced immediately as described below.

Intein cleavage and purification of TglASec, TglAHcy, TglADCys, and TglAPen. For each peptide, a column containing 30 mL of chitin resin was loaded with 50 mL oxygen-free cleavage buffer (50 mM HEPES, 200 mM NaCl, 1 mM EDTA, 75 mM 4-mercaptophenylacetic acid,[4] pH 8.0) containing 1 mM PMSF, 1 µM leupeptin, and one of the following specific cleavage mixtures: TglASec, 10 mM

L-selenocystine, 50 mM DTT, 30 mM HEPES free acid (final pH 7.5); TglAHcy, 12.5 mM L-homocystine, 30 mM DTT (final pH 8.0); TglADCys, 50 mM D-cysteine, 5 mM DTT; TglAPen, 50 mM L-penicillamine. Approx. 25 mL of flowthrough was discarded and the column was incubated on a nutator at room temperature in the anaerobic chamber for 14-15 h. The column was drained and washed with 17 mL of fusion buffer, and the combined flowthrough (40-45 mL) containing the crude peptide was retained. Approx. 20 mL of the crude mixture was stored at -80 °C and the remaining 20-25 mL was desalted using a 1-g Supel-Select HLB SPE cartridge (Sigma-Aldrich) and nitrogen-sparged solvents. Lyophilized elution fractions were suspended in nitrogen-sparged water containing 5-10 mM DTT, adjusted to pH 6-7 with NH_4OH to dissolve, filtered, and purified by HPLC as described for His_6-TglACys except with a modified gradient: initial equilibrium at 3% B was held for 4 min after sample injection, followed by a 23-minute linear gradient of 3-75% B. TglASec eluted from 19.8-20.3 min, TglAHcy from 19.4-19.9 min, TglADCys from 19.3-19.8 min, and TglAPen from 19.6-20.1 min. Lyophilized fractions containing pure peptide as judged by MALDI-TOF MS were redissolved in water, adjusted to pH 6-8 with NH_4OH/acetic acid, and stored at −80 °C. An iodoacetamide-alkylated, GluC-digested sample of TglASec was further characterized by ESI-MS using a Thermo Q Exactive Hybrid Quadrupole-Orbitrap instrument to confirm the presence of Se by its distinctive isotope distribution (**Figure S7**). The TglASec sample was treated with 100 mM NaOH to remove a persistent impurity formed during the ligation process; after 30 min, the impurity hydrolyzed completely to form TglA (**Figure S5a**). Following hydrolysis, the sample was neutralized to pH 6-8 with HCl and used for *in vitro* reactions without additional purification. The assay buffer (50 mM Na_2HPO_4, 300 mM NaCl, 10% [v/v] glycerol, pH 7.6) assured that the conditions of all enzymatic assays were at the same pH.

Concentration determination of purified peptides. Since TglACys lacks Trp and Tyr residues and thus has negligible absorbance at 280 nm, concentrations of His_6-TglACys, TglASec (before alkaline hydrolysis), TglAHcy, TglADCys, and TglAPen were estimated by reduction of 5,5′-dithiobis(2-nitrobenzoic acid) (DTNB). Briefly, stock solutions of peptides (200-800 µM) were combined with equal volumes of DTNB stock solution (5 mM in 100 mM KH_2PO_4/K_2HPO_4 pH 7.5 + 0.25 mM EDTA), and 1-mm absorbances at 409-410 nm were compared with absorbances of samples derived from standard curves of 0.1-1 mM D-cysteine (for TglADCys), L-penicillamine (for TglAPen), or β-mercaptoethanol (for all other peptides). Concentrations determined by this method do not account for oxidized peptide species, such as disulfide or diselenide dimers, which are not expected to be competent substrates for TglHI.

MALDI-TOF MS analysis. Approx. 0.5-1 µL of desalted sample was spotted on a MALDI plate, mixed with 0.5-1 µL of Super DHB matrix (50 mg/mL in 50% [v/v] MeCN + 0.1% [v/v] TFA), and allowed to dry. Spots were analyzed on a Bruker UltraFleXtreme MALDI-TOF/TOF spectrometer in positive ion mode with reflector geometry.

Native mass spectrometry analysis. To prepare a sample of His_6-TglACys – CH_2 bound to His_6TglHI, His_6-TglACys and His_6-TglHI were coexpressed in *E. coli* BL21(DE3) cells transformed with both plasmids and the overexpressed proteins were purified by Ni-NTA resin similar to the procedure for purifying His_6-TglHI. The eluted protein was further purified by size exclusion chromatography using a Superdex 200 Increase column and 50 mM HEPES, 300 mM NaCl, 10 % glycerol, pH 7.6 as the mobile

phase. The major fraction was concentrated using a 30 kDa molecular weight cutoff (MWCO) filter to 1 mg/mL and desalted twice into 25 mM ammonium acetate using two 0.5 mL 40 kDa MWCO Zeba spin columns. To prepare a sample of TglA bound to His_6-TglHI, 50 µM synthetic TglA was incubated with 50 µM His_6-TglHI at room temperature for 2 h in assay buffer. The protein solution was desalted twice using 50 mM ammonium acetate employing two 0.5 mL 40 kDa MWCO Zeba spin columns. Native MS analysis was performed on a Q Exactive UHMR Hybrid Quadrupole-Orbitrap MS system with an Advion TriVersa NanoMate nanoelectrospray ion source. MS parameters were as follows: resolution 12500, scan range 500.0 - 12000.0 m/z, microscans 10, maximum injection time 50, advance gain control target 5×10^4, capillary temperature 200 °C, trapping gas pressure 3, in-source trapping on, desolvation voltage –150 V, S-lens RF level 200. Data analysis was performed using BioPharma Finder software and the ReSpect algorithm was chosen for deconvolution.

Sequence of codon-optimized synthetic gene for TglACys[38-45A]

5′-
CAAAACACAAACTATAATTATAAAACAATATAAATAAAAATAAAGGCGTAATACGACTCACTATAGGGT
TAACTTTAACAAGGAGAAAAACATGGGTCAACCGAACGTGCAGAGCGTTGAAAACCAGCAGGCGTCAGG
CGATGTTAAAGATCTTGAAAACACTCCGCAGGCCACAGAAGAAGCGTTATTTGAGGAGTTTGACGCAGC
AGCGGCGGCAGCGGCAGCGTCCAAAGTGTTTGCGTGCTAAGCTTCGAAAACAAACACAAATAATAACAA
AATAAAAATTAACAAAAACTTAAC-3′
The sequence encoding Ala residues at positions 38-45 is shown in yellow.

Table S1. Sequences of synthetic TglA peptides. Ala substitutions are shown in red font. If an Ala was already present in the native sequence, black font is used.

Peptide	Sequence
TglA Wt	MGQPNVQSVENQQASGDVKDLENTPQATEEALFEE**FDLD**DIEVIESKVFA
TglA[6-13A]	MGQPN**AAAAAAAA**ASGDVKDLENTPQATEEALFEE**FDLD**DIEVIESKVFA
TglA[10-17A]	MGQPNVQSV**AAAA**A**AAA**VKDLENTPQATEEALFEE**FDLD**DIEVIESKVFA
TglA[14-21A]	MGQPNVQSVENQQA**AAAAAAA**ENTPQATEEALFEE**FDLD**DIEVIESKVFA
TglA[18-25A]	MGQPNVQSVENQQASGD**AAAAAAAA**QATEEALFEE**FDLD**DIEVIESKVFA
TglA[22-29A]	MGQPNVQSVENQQASGDVKDL**AAAAA**A**AA**EALFEE**FDLD**DIEVIESKVFA
TglA[26-33A]	MGQPNVQSVENQQASGDVKDLENTP**A**A**AAA**A**AA**EE**FDLD**DIEVIESKVFA
TglA[30-37A]	MGQPNVQSVENQQASGDVKDLENTPQATE**A**A**AAAAAA****LD**DIEVIESKVFA
TglA[34-41A]	MGQPNVQSVENQQASGDVKDLENTPQATEEALF**AAAAAAAA**EVIESKVFA
TglA[38-45A]	MGQPNVQSVENQQASGDVKDLENTPQATEEALFEE**FD****AAAAAAAA**SKVFA

Table S2. Observed and theoretical *m/z* values for peptide ions in TglB-TglHI coupled assays. Color-coded as in Figure 2.

	Substrate $[M+H]^+$ *m/z*			Product $[M+H]^+$ *m/z*		
**Peptide**	**theoretical**	**observed**	**Δ (Da)**	**theoretical**	**observed**	**Δ (Da)**
TglA Wt	5553.601	5553.147	-0.454	5642.595	5642.158	-0.437
TglA[6-13A]	5209.468	5209.454	-0.014	5298.462	5298.494	0.032
TglA[10-17A]	5292.578	5292.526	-0.052	5381.572	5381.506	-0.066
TglA[14-21A]	5336.506	5336.657	0.151	5425.500	5425.485	-0.015
TglA[18-25A]	5225.438	5225.427	-0.011	5314.431	5314.588	0.157
TglA[22-29A]	5251.526	5251.684	0.158	5340.520	5340.806	0.286
TglA[26-33A]	5232.480	5232.630	0.150	5321.473	5321.936	0.463
TglA[30-37A]	5141.486	5141.784	0.298	5244.495	5245.202	0.707
TglA[34-41A]	5145.496	5144.876	-0.620	5248.505	5248.021	-0.484
TglA[38-45A]	5195.439	5195.761	0.322	5195.439	5196.006	0.567

Table S3. Sequences of short TglACys peptides generated by IVT. Differences from the native sequence are shown in red. The FDLD motif is shown in blue. Red dash (–) means deletion of residues at these positions.

Name	Sequence	Name	Sequence
12mer	MDIEVIESKVFAC		
13mer	MDDIEVIESKVFAC	19mer-ΔS46	MFEEFDLDDIEVIE-KVFAC
14mer	MLDDIEVIESKVFAC	19mer-ins50A	MFEEFDLDDIEVIESKVFAAC
15mer	MDLDDIEVIESKVFAC	19mer-ins49A	MFEEFDLDDIEVIESKVAFAC
16mer	MFDLDDIEVIESKVFAC	19mer-ins48A	MFEEFDLDDIEVIESKAVFAC
17mer	MEFDLDDIEVIESKVFAC	19mer-ins47A	MFEEFDLDDIEVIESAKVFAC
18mer	MEEFDLDDIEVIESKVFAC	19mer-ins46A	MFEEFDLDDIEVIEASKVFAC
19mer	MFEEFDLDDIEVIESKVFAC	19mer-ΔF49ΔA50	MFEEFDLDDIEVIESKV--C
20mer	MLFEEFDLDDIEVIESKVFAC	19mer-ΔV48ΔF49	MFEEFDLDDIEVIESK--AC
21mer	MALFEEFDLDDIEVIESKVFAC	19mer-ΔK47ΔV48	MFEEFDLDDIEVIES--FAC
22mer	MEALFEEFDLDDIEVIESKVFAC	19mer-ΔS46ΔK47	MFEEFDLDDIEVIE--VFAC
23mer	MEEALFEEFDLDDIEVIESKVFAC	19mer-ΔE45ΔS46	MFEEFDLDDIEVI--KVFAC
19mer-AALD	MFEEAALDDIEVIESKVFAC	19mer-F49A	MFEEFDLDDIEVIESKVAAC
19mer-FAAD	MFEEFAADDIEVIESKVFAC	19mer-V48A	MFEEFDLDDIEVIESKAFAC
19mer-ADAD	MFEEADADDIEVIESKVFAC	19mer-K47A	MFEEFDLDDIEVIESAVFAC
19mer-FALA	MFEEFALADIEVIESKVFAC	19mer-S46A	MFEEFDLDDIEVIEAKVFAC
19mer-ADLA	MFEEADLADIEVIESKVFAC	19mer-E45A	MFEEFDLDDIEVIASKVFAC
19mer-FDAA	MFEEFDAADIEVIESKVFAC	19mer-I44A	MFEEFDLDDIEVAESKVFAC
19mer-AAAA	MFEEAAAADIEVIESKVFAC	19mer-V43A	MFEEFDLDDIEAIESKVFAC
19mer-A50V	MFEEFDLDDIEVIESKVFVC	19mer-E42A	MFEEFDLDDIAVIESKVFAC
19mer-A50F	MFEEFDLDDIEVIESKVFFC	19mer-I41A	MFEEFDLDDAEVIESKVFAC
19mer-A50G	MFEEFDLDDIEVIESKVFGC	19mer-D40A	MFEEFDLDAIEVIESKVFAC
19mer-A50S	MFEEFDLDDIEVIESKVFSC	19mer-D39A	MFEEFDLADIEVIESKVFAC
19mer-A50K	MFEEFDLDDIEVIESKVFKC	19mer-L38A	MFEEFDADDIEVIESKVFAC
19mer-A50D	MFEEFDLDDIEVIESKVFDC	19mer-D37A	MFEEFALDDIEVIESKVFAC
19mer-ΔA50	MFEEFDLDDIEVIESKVF-C	19mer-F36A	MFEEADLDDIEVIESKVFAC
19mer-ΔF49	MFEEFDLDDIEVIESKV-AC	19mer-E35A	MFEAFDLDDIEVIESKVFAC
19mer-ΔV48	MFEEFDLDDIEVIESK-FAC	19mer-E34A	MFAEFDLDDIEVIESKVFAC
19mer-ΔK47	MFEEFDLDDIEVIES-VFAC	19mer-F33A	MAEEFDLDDIEVIESKVFAC

Table S4. Oligonucleotide sequences.

Name	Sequence (5′-3′)
TglACys-IVT-F	GGCGTAATACGACTCACTATAGG
TglACys-IVT-R	cgaagcttagcacgcaaac
TglACys-F1	GGCGTAATACGACTCACTATAGGGTTAACTTTAACAAGGAGAAAAAC
TglACys-F2	GGCGTAATACGACTCACTATAGGGTTAAC
13mer-R1	TGGACTCAATTACCTCGATGTCGTCCATGTTTTTCTCCTTGTTAAAGTTAACCC
14mer-R1	GGACTCAATTACCTCGATGTCGTCAAGCATGTTTTTCTCCTTGTTAAAGTTAACCC
15mer-R1	GACTCAATTACCTCGATGTCGTCAAGGTCCATGTTTTTCTCCTTGTTAAAGTTAACCC
16mer-R1	CTCAATTACCTCGATGTCGTCAAGGTCAAACATGTTTTTCTCCTTGTTAAAGTTAACCC
17mer-R1	TACCTCGATGTCGTCAAGGTCAAACTCCATGTTTTTCTCCTTGTTAAAGTTAACCC
18mer-R1	TACCTCGATGTCGTCAAGGTCAAACTCCTCCATGTTTTTCTCCTTGTTAAAGTTAACCC
19mer-R1	CCTCGATGTCGTCAAGGTCAAACTCCTCAAACATGTTTTTCTCCTTGTTAAAGTTAACC
20mer-R1	TCGATGTCGTCAAGGTCAAACTCCTCAAAGAGCATGTTTTTCTCCTTGTTAAAGTTAAC
21mer-R1	GATGTCGTCAAGGTCAAACTCCTCAAAGAGTGCCATGTTTTTCTCCTTGTTAAAGTTAAC
22mer-R1	GTCGTCAAGGTCAAACTCCTCAAAGAGTGCCTCCATGTTTTTCTCCTTGTTAAAGTTAAC
23mer-R1b	GTCAAGGTCAAACTCCTCAAAGAGTGCCTCCTCCATGTTTTTCTCCTTGTTAAAGTTAAC
WT-R2	CGAAGCTTAGCACGCAAACACTTTGGACTCAATTACCTCGATGTCGTCAAGGTCAAACTC
13-16mer-R2.2	CGAAGCTTAGCACGCAAACACTTTGGACTCAATTACCTCGATGTCGTC
12mer-F	GGCGTAATACGACTCACTATAGGGTTAACTTTAACAAGGAGAAAAACATGGACATCGAG
12mer-R	CGAAGCTTAGCACGCAAACACTTTGGACTCAATTACCTCGATGTCCATGTTTTTCTCC
AALD-R1	CCTCGATGTCGTCAAGtgccgcCTCCTCAAACATGTTTTTCTCCTTGTTAAAGTTAACC
FAAD-R1	CCTCGATGTCGTCtgctgcAAACTCCTCAAACATGTTTTTCTCCTTGTTAAAGTTAACC
ADAD-R1	CCTCGATGTCGTCtgcGTCtgcCTCCTCAAACATGTTTTTCTCCTTGTTAAAGTTAACC
FALA-R1	CCTCGATGTCtgcAAGtgcAAACTCCTCAAACATGTTTTTCTCCTTGTTAAAGTTAACC
ADLA-R1	CCTCGATGTCtgcAAGGTCtgcCTCCTCAAACATGTTTTTCTCCTTGTTAAAGTTAACC
FDAA-R1	CCTCGATGTCtgctgcGTCAAACTCCTCAAACATGTTTTTCTCCTTGTTAAAGTTAACC
AAAA-R1	CCTCGATGTCtgctgccgctgcCTCCTCAAACATGTTTTTCTCCTTGTTAAAGTTAACC
AALD-R2.2	CGAAGCTTAGCACGCAAACACTTTGGACTCAATTACCTCGATGTCGTCAAGtgctg
FAAD-R2	CGAAGCTTAGCACGCAAACACTTTGGACTCAATTACCTCGATGTCGTCtgctgcAAACTC
ADAD-R2.2	CGAAGCTTAGCACGCAAACACTTTGGACTCAATTACCTCGATGTCGTCtgcGTC
FALA-R2	CGAAGCTTAGCACGCAAACACTTTGGACTCAATTACCTCGATGTCtgcAAGtgcAAACTC
ADLA-R2.2	CGAAGCTTAGCACGCAAACACTTTGGACTCAATTACCTCGATGTCtgcAAGGTCtg
FDAA-R2	CGAAGCTTAGCACGCAAACACTTTGGACTCAATTACCTCGATGTCtgctgcGTCAAACTC
AAAA-R2.2	CGAAGCTTAGCACGCAAACACTTTGGACTCAATTACCTCGATGTCtgctgccg
A50V-R2	CGAAGCTTAGCAtacAAACACTTTGGACTCAATTACCTCGATGTCGTCAAGGTCAAACTC
A50F-R2	CGAAGCTTAGCAaaaAAACACTTTGGACTCAATTACCTCGATGTCGTCAAGGTCAAACTC
A50G-R2	CGAAGCTTAGCAgccAAACACTTTGGACTCAATTACCTCGATGTCGTCAAGGTCAAACTC
A50S-R2	CGAAGCTTAGCAggaAAACACTTTGGACTCAATTACCTCGATGTCGTCAAGGTCAAACTC
A50K-R2	CGAAGCTTAGCAtttAAACACTTTGGACTCAATTACCTCGATGTCGTCAAGGTCAAACTC
A50D-R2	CGAAGCTTAGCAgtcAAACACTTTGGACTCAATTACCTCGATGTCGTCAAGGTCAAACTC
delA50-R2	CGAAGCTTAGCAAAACACTTTGGACTCAATTACCTCGATGTCGTCAAGGTCAAACTC
delF49-R2	CGAAGCTTAGCACGCCACTTTGGACTCAATTACCTCGATGTCGTCAAGGTCAAACTC
delV48-R2	CGAAGCTTAGCACGCAAATTTGGACTCAATTACCTCGATGTCGTCAAGGTCAAACTC
delK47-R2	CGAAGCTTAGCACGCAAACACGGACTCAATTACCTCGATGTCGTCAAGGTCAAACTC
delS46-R2	CGAAGCTTAGCACGCAAACACTTTCTCAATTACCTCGATGTCGTCAAGGTCAAACTC
ins50A-R2	CGAAGCTTAGCACGCtgcAAACACTTTGGACTCAATTACCTCGATGTCGTCAAGGTCAAA
ins49A-R2	CGAAGCTTAGCACGCAAAtgcCACTTTGGACTCAATTACCTCGATGTCGTCAAGGTCAAA
ins48A-R2	CGAAGCTTAGCACGCAAACACtgcTTTGGACTCAATTACCTCGATGTCGTCAAGGTCAAA
ins47A-R2	CGAAGCTTAGCACGCAAACACTTTtgcGGACTCAATTACCTCGATGTCGTCAAGGTCAAA
ins46A-R2	CGAAGCTTAGCACGCAAACACTTTGGAtgcCTCAATTACCTCGATGTCGTCAAGGTCAAA

(continued on next page)

Table S4, cont.

Name	Sequence (5′-3′)
delFA4950-R2	CGAAGCTTAGCACACTTTGGACTCAATTACCTCGATGTCGTCAAGGTCAAACTC
delVF4849-R2	CGAAGCTTAGCACGCTTTGGACTCAATTACCTCGATGTCGTCAAGGTCAAACTC
delKV4748-R2	CGAAGCTTAGCACGCAAAGGACTCAATTACCTCGATGTCGTCAAGGTCAAACTC
delSK4647-R2	CGAAGCTTAGCACGCAAACACCTCAATTACCTCGATGTCGTCAAGGTCAAACTC
delES4546-R2	CGAAGCTTAGCACGCAAACACTTTAATTACCTCGATGTCGTCAAGGTCAAACTC
F49A-R2	CGAAGCTTAGCACGCtgcCACTTTGGACTCAATTACCTCGATGTCGTCAAGGTCAAACTC
V48A-R2	CGAAGCTTAGCACGCAAAtgcTTTGGACTCAATTACCTCGATGTCGTCAAGGTCAAACTC
K47A-R2	CGAAGCTTAGCACGCAAACACtgcGGACTCAATTACCTCGATGTCGTCAAGGTCAAACTC
S46A-R2	CGAAGCTTAGCACGCAAACACTTTtgcCTCAATTACCTCGATGTCGTCAAGGTCAAACTC
E45A-R2	CGAAGCTTAGCACGCAAACACTTTGGAtgcAATTACCTCGATGTCGTCAAGGTCAAACTC
I44A-R2	CGAAGCTTAGCACGCAAACACTTTGGACTCtgcTACCTCGATGTCGTCAAGGTCAAACTC
V43A-R2	CGAAGCTTAGCACGCAAACACTTTGGACTCAATTgCCTCGATGTCGTCAAGGTCAAACTC
E42A-R1	CtgcGATGTCGTCAAGGTCAAACTCCTCAAACATGTTTTTCTCCTTGTTAAAGTTAACC
I41A-R1	CCTCtgcGTCGTCAAGGTCAAACTCCTCAAACATGTTTTTCTCCTTGTTAAAGTTAACC
D40A-R1	CCTCGATtgcGTCAAGGTCAAACTCCTCAAACATGTTTTTCTCCTTGTTAAAGTTAACC
D39A-R1	CCTCGATGTCtgcAAGGTCAAACTCCTCAAACATGTTTTTCTCCTTGTTAAAGTTAACC
L38A-R1	CCTCGATGTCGTCtgcGTCAAACTCCTCAAACATGTTTTTCTCCTTGTTAAAGTTAACC
D37A-R1	CCTCGATGTCGTCAAGtgcAAACTCCTCAAACATGTTTTTCTCCTTGTTAAAGTTAACC
F36A-R1	CCTCGATGTCGTCAAGGTCtgcCTCCTCAAACATGTTTTTCTCCTTGTTAAAGTTAACC
E35A-R1	CCTCGATGTCGTCAAGGTCAAAtgcCTCAAACATGTTTTTCTCCTTGTTAAAGTTAACC
E34A-R1	CCTCGATGTCGTCAAGGTCAAACTCtgcAAACATGTTTTTCTCCTTGTTAAAGTTAACC
F33A-R1	CCTCGATGTCGTCAAGGTCAAACTCCTCtgcCATGTTTTTCTCCTTGTTAAAGTTAACC
E42A-R2	CGAAGCTTAGCACGCAAACACTTTGGACTCAATTACtgcGATGTCGTCAAGGTCAAACTC
I41A-R2	CGAAGCTTAGCACGCAAACACTTTGGACTCAATTACCTCtgcGTCGTCAAGGTCAAACTC
D40A-R2	CGAAGCTTAGCACGCAAACACTTTGGACTCAATTACCTCGATtgcGTCAAGGTCAAACTC
D39A-R2	CGAAGCTTAGCACGCAAACACTTTGGACTCAATTACCTCGATGTCtgcAAGGTCAAACTC
L38A-R2	CGAAGCTTAGCACGCAAACACTTTGGACTCAATTACCTCGATGTCGTCtgcGTCAAACTC
D37A-R2	CGAAGCTTAGCACGCAAACACTTTGGACTCAATTACCTCGATGTCGTCAAGtgcAAACTC
F36A-R2.2	CGAAGCTTAGCACGCAAACACTTTGGACTCAATTACCTCGATGTCGTCAAGGTCtg
E35A-R2	CGAAGCTTAGCACGCAAACACTTTGGACTCAATTACCTCGATGTCGTCAAGGTCAAAtgc

Table S5. PCR conditions for construction of TglACys fragment IVT templates.

Name	PCR1 fwd primer	PCR1 rev primer	PCR1 anneal (°C)	PCR2 fwd primer	PCR2 rev primer	PCR2 anneal (°C)
12mer				12mer-F	12mer-R	56
13mer	TglACys-F1	13mer-R1	54	TglACys-F2	13-16mer-R2.2	61
14mer	TglACys-F1	14mer-R1	54	TglACys-F2	13-16mer-R2.2	61
15mer	TglACys-F1	15mer-R1	54	TglACys-F2	13-16mer-R2.2	61
16mer	TglACys-F1	16mer-R1	54	TglACys-F2	13-16mer-R2.2	61
17mer	TglACys-F1	17mer-R1	54	TglACys-F2	WT-R2	61
18mer	TglACys-F1	18mer-R1	54	TglACys-F2	WT-R2	61
19mer	TglACys-F1	19mer-R1	52	TglACys-F2	WT-R2	61
20mer	TglACys-F1	20mer-R1	49	TglACys-F2	WT-R2	59
21mer	TglACys-F1	21mer-R1	49	TglACys-F2	WT-R2	55
22mer	TglACys-F1	22mer-R1	49	TglACys-F2	WT-R2	52
23merB	TglACys-F1	23mer-R1b	49	TglACys-F2	WT-R2	45
19mer-aald	TglACys-F1	AALD-R1	52	TglACys-F2	AALD-R2.2	61
19mer-faad	TglACys-F1	FAAD-R1	52	TglACys-F2	FAAD-R2	61
19mer-adad	TglACys-F1	ADAD-R1	52	TglACys-F2	ADAD-R2.2	61
19mer-fala	TglACys-F1	FALA-R1	52	TglACys-F2	FALA-R2	61
19mer-adla	TglACys-F1	ADLA-R1	52	TglACys-F2	ADLA-R2.2	61
19mer-fdaa	TglACys-F1	FDAA-R1	52	TglACys-F2	FDAA-R2	61
19mer-aaaa	TglACys-F1	AAAA-R1	52	TglACys-F2	AAAA-R2.2	61
19mer-a50v	TglACys-F1	19mer-R1	52	TglACys-F2	A50V-R2	61
19mer-a50f	TglACys-F1	19mer-R1	52	TglACys-F2	A50F-R2	61
19mer-a50g	TglACys-F1	19mer-R1	52	TglACys-F2	A50G-R2	61
19mer-a50s	TglACys-F1	19mer-R1	52	TglACys-F2	A50S-R2	61
19mer-a50k	TglACys-F1	19mer-R1	52	TglACys-F2	A50K-R2	61
19mer-a50d	TglACys-F1	19mer-R1	52	TglACys-F2	A50D-R2	61
19mer-Δa50	TglACys-F1	19mer-R1	52	TglACys-F2	delA50-R2	61
19mer-Δf49	TglACys-F1	19mer-R1	52	TglACys-F2	delF49-R2	61
19mer-Δv48	TglACys-F1	19mer-R1	52	TglACys-F2	delV48-R2	61
19mer-Δk47	TglACys-F1	19mer-R1	52	TglACys-F2	delK47-R2	61
19mer-Δs46	TglACys-F1	19mer-R1	52	TglACys-F2	delS46-R2	61
19mer-ins50a	TglACys-F1	19mer-R1	52	TglACys-F2	ins50A-R2	61
19mer-ins49a	TglACys-F1	19mer-R1	52	TglACys-F2	ins49A-R2	61
19mer-ins48a	TglACys-F1	19mer-R1	52	TglACys-F2	ins48A-R2	61
19mer-ins47a	TglACys-F1	19mer-R1	52	TglACys-F2	ins47A-R2	61
19mer-ins46a	TglACys-F1	19mer-R1	52	TglACys-F2	ins46A-R2	61
19mer-Δfa4950	TglACys-F1	19mer-R1	52	TglACys-F2	delFA4950-R2	61
19mer-Δvf4849	TglACys-F1	19mer-R1	52	TglACys-F2	delVF4849-R2	61
19mer-Δkv4748	TglACys-F1	19mer-R1	52	TglACys-F2	delKV4748-R2	61
19mer-Δsk4647	TglACys-F1	19mer-R1	52	TglACys-F2	delSK4647-R2	61
19mer-Δes4546	TglACys-F1	19mer-R1	52	TglACys-F2	delES4546-R2	61
19mer-f49a	TglACys-F1	19mer-R1	52	TglACys-F2	F49A-R2	61
19mer-v48a	TglACys-F1	19mer-R1	52	TglACys-F2	V48A-R2	61
19mer-k47a	TglACys-F1	19mer-R1	52	TglACys-F2	K47A-R2	61
19mer-s46a	TglACys-F1	19mer-R1	52	TglACys-F2	S46A-R2	61
19mer-e45a	TglACys-F1	19mer-R1	52	TglACys-F2	E45A-R2	61

(continued on next page)

Table S5, cont.

Name	PCR1 fwd primer	PCR1 rev primer	PCR1 anneal (°C)	PCR2 fwd primer	PCR2 rev primer	PCR2 anneal (°C)
19mer-i44a	TglACys-F1	19mer-R1	52	TglACys-F2	I44A-R2	61
19mer-v43a	TglACys-F1	19mer-R1	52	TglACys-F2	V43A-R2	61
19mer-e42a	TglACys-F1	E42A-R1	52	TglACys-F2	E42A-R2	61
19mer-i41a	TglACys-F1	I41A-R1	52	TglACys-F2	I41A-R2	61
19mer-d40a	TglACys-F1	D40A-R1	52	TglACys-F2	D40A-R2	61
19mer-d39a	TglACys-F1	D39A-R1	52	TglACys-F2	D39A-R2	61
19mer-l38a	TglACys-F1	L38A-R1	52	TglACys-F2	L38A-R2	61
19mer-d37a	TglACys-F1	D37A-R1	52	TglACys-F2	D37A-R2	61
19mer-f36a	TglACys-F1	F36A-R1	52	TglACys-F2	F36A-R2.2	59
19mer-e35a	TglACys-F1	E35A-R1	52	TglACys-F2	E35A-R2	61
19mer-e34a	TglACys-F1	E34A-R1	52	TglACys-F2	WT-R2	61
19mer-f33a	TglACys-F1	F33A-R1	52	TglACys-F2	WT-R2	61

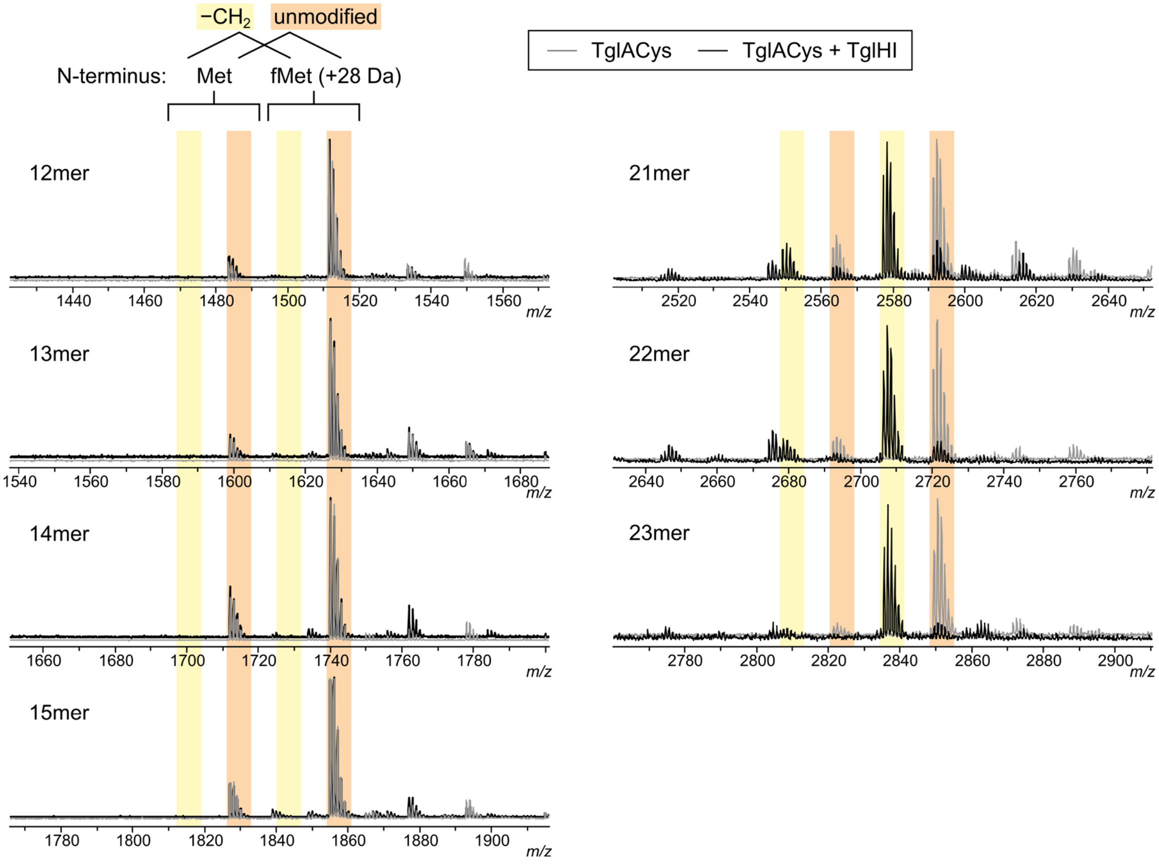

Figure S1. TglHI activity toward TglACys fragments that are shorter (left) and longer (right) than the 19mer discussed in the main text. MALDI-TOF mass spectra depict IVT-generated C-terminal fragments of TglACys (gray) and the products of 2-h *in vitro* reactions with TglHI (black).

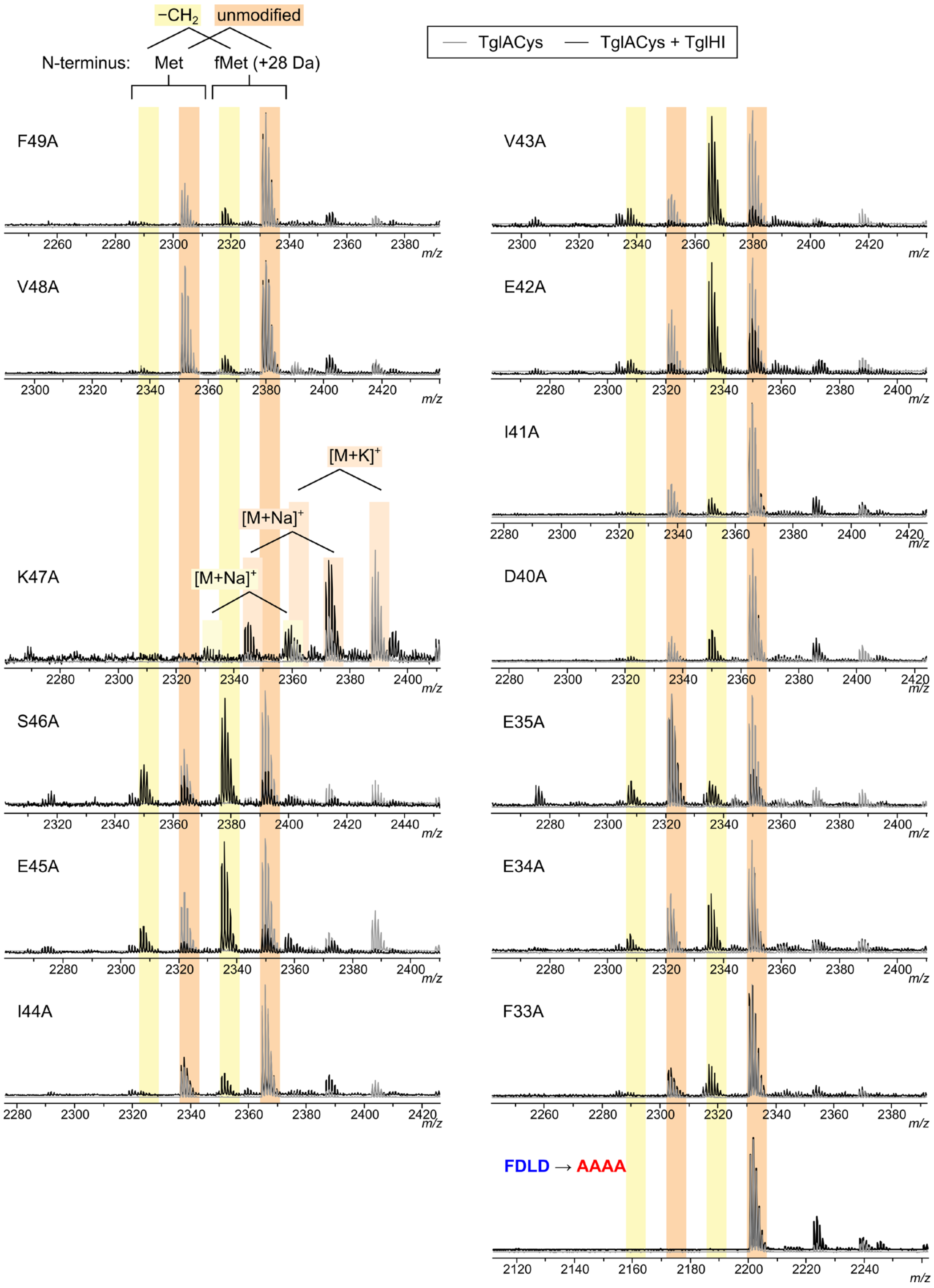

Figure S2. TglHI activity toward 19mer TglACys peptides with alanine substitutions. MALDI-TOF mass spectra depict IVT-generated 19mer C-terminal TglACys fragments (gray) and the products of 2 h *in vitro* reactions with TglHI (black). Amino acid residue numbers are based on the full-length TglACys peptide.

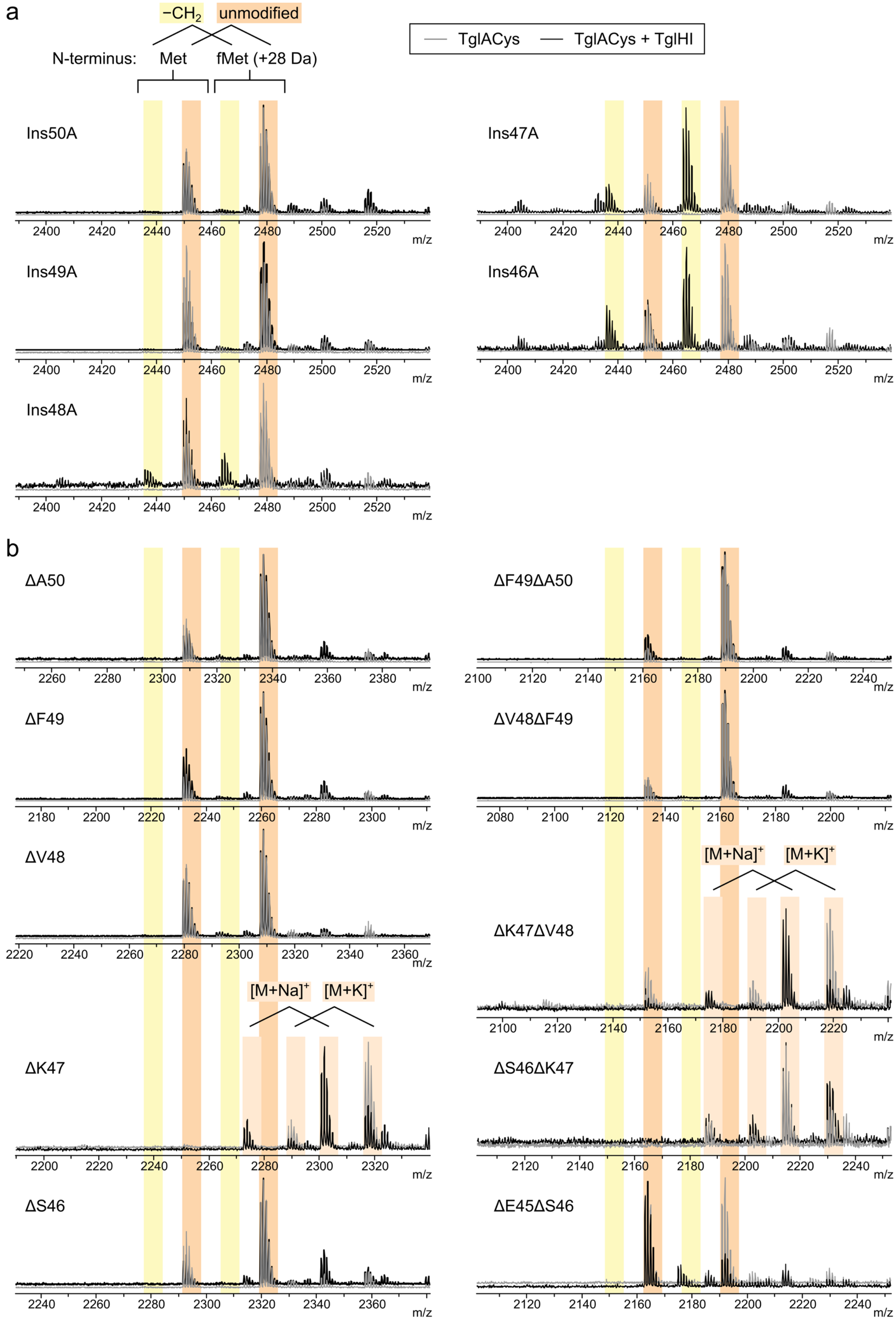
a
−CH_2
unmodified
N-terminus:
Met
fMet (+28 Da)
TglACys
TglACys + TglHI
Ins50A
Ins49A
Ins48A
Ins47A
Ins46A
m/z
b
ΔA50
ΔF49
ΔV48
ΔK47
ΔS46
ΔF49ΔA50
ΔV48ΔF49
ΔK47ΔV48
ΔS46ΔK47
ΔE45ΔS46
$[M+Na]^+$
$[M+K]^+$

Figure S3 (previous page). TglHI activity toward 19mer TglACys peptides with insertions and deletions. Pairs of MALDI-TOF mass spectra depict IVT-generated C-terminal fragments of TglACys before TglHI addition (gray) and the products of 2 h reactions with TglHI (black). **(a)** Alanine insertions at the five positions before the C-terminal Cys. **(b)** Single and double deletions at the five positions before the C-terminal Cys. Amino acid residue numbers are based on the full-length TglACys peptide.

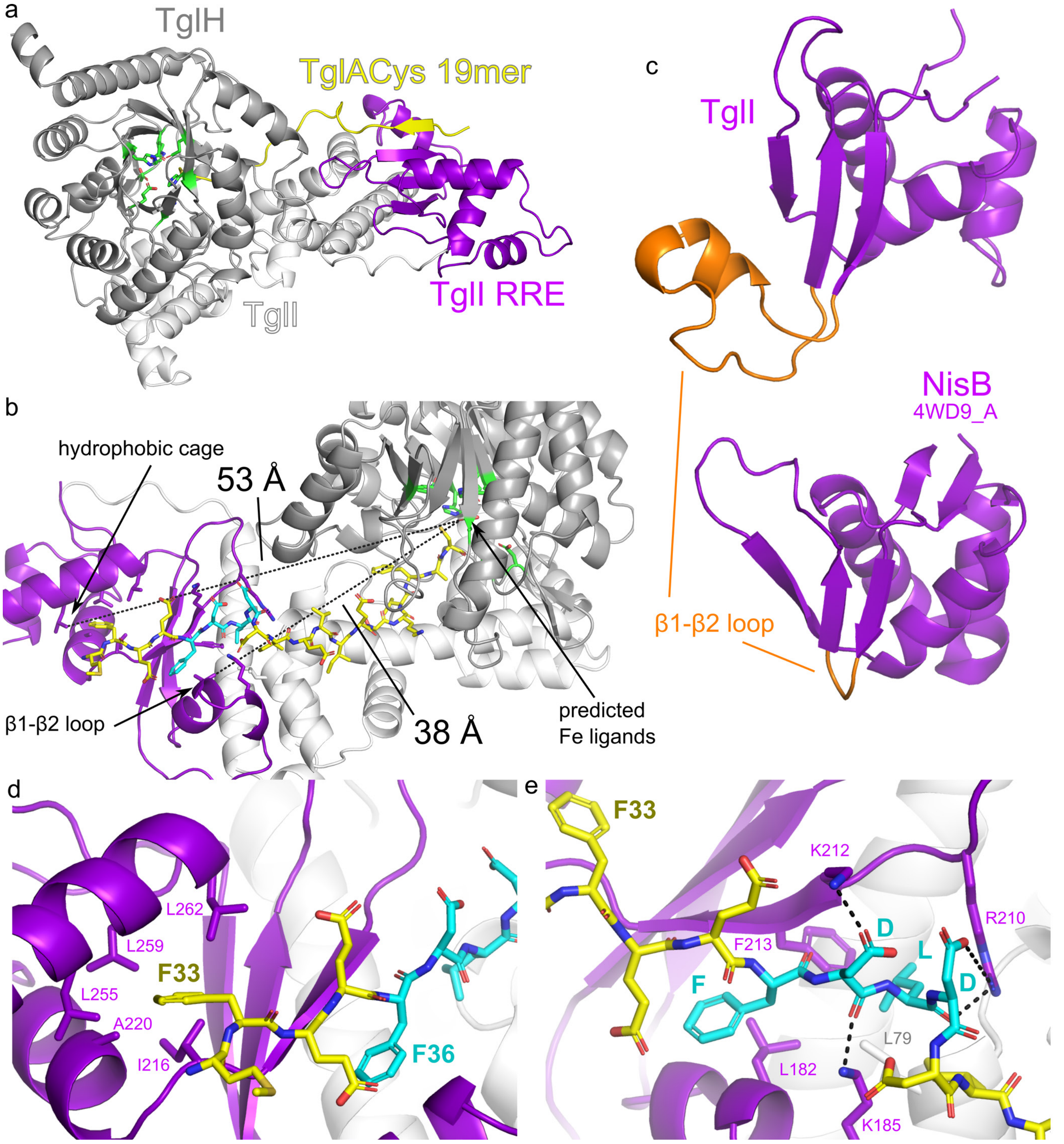

Figure S4 (previous page). Predicted structure of the TglHI complex with the TglACys 19mer using AlphaFold-Multimer, a protein complex structure prediction algorithm incorporating AlphaFold2.[5, 6] The predicted structure of the TglHI complex is nearly identical in the absence of TglACys and in the presence of the full-length peptide. **(a)** Overall architecture of the complex. The TglH and TglI polypeptides are colored in gray and white, respectively. The eight predicted iron ligands in TglH are colored in green and the predicted RiPP recognition element (RRE, residue 162-269) domain of TglI is colored in purple. TglACys is shown in yellow. **(b)** Predicted binding mode of TglACys, colored as in (a) except with the F[N/D]LD motif in cyan. The motif interacts with an extended loop between strands β1 and β2 of the RRE rather than with the hydrophobic cage that binds the Phe of the corresponding motif in the NisB/NisA structure. Distances between the catalytic site of TglH and the RRE domain of TglI were estimated using the minimum distance between side chain atoms of TglH residue Glu258 (putative iron ligand) and TglI residue Leu255 (hydrophobic cage) or Leu182 (β1-β2 loop). **(c)** The β1-β2 loop of the RRE, highlighted in orange, is much longer in the TglHI-TglACys model than in the crystal structure of NisB (PDB ID: 4WD9, chain A); the latter loop is similar in other structurally characterized RREs. **(d)** Interaction of the TglI hydrophobic cage with TglACys, colored as in (b); the cage is predicted to interact with Phe33 of TglACys rather than Phe36 of the F[N/D]LD motif. **(e)** Predicted binding mode of the TglACys F[N/D]LD motif to TglI. Polar interactions with TglI side chains are shown as dashed lines; hydrogen bonds forming an antiparallel β sheet structure of TglA with β3 of the RRE are omitted for clarity. Leu79 of TglI, which is not part of the canonical RRE, is predicted to form part of a hydrophobic pocket for the Leu38 residue in the F[N/D]LD motif.

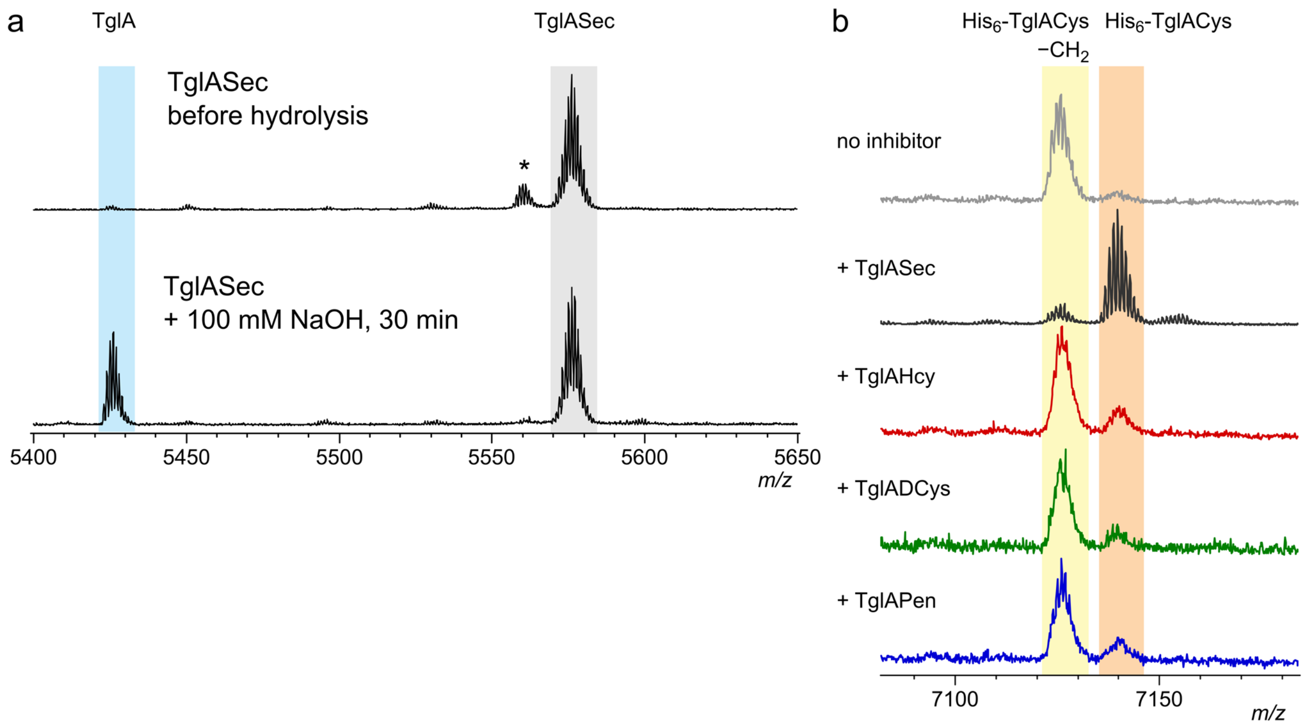

Figure S5. Unnatural analogues of TglACys. **(a)** MALDI-TOF mass spectra of TglASec before and after hydrolysis with 100 mM NaOH for 30 min. The asterisk marks an unknown impurity. We suspect the impurity to be a thioester or similar species based on its base-lability; however, we did not further investigate its identity. **(b)** MALDI-TOF mass spectra of 50 µM His_6-TglACys after 30 min incubation with 5 µM TglHI in the absence of inhibitor or in the presence of 50 µM TglASec, TglAHcy, TglADCys, or TglAPen. Only TglASec inhibits modification of His_6-TglACys.

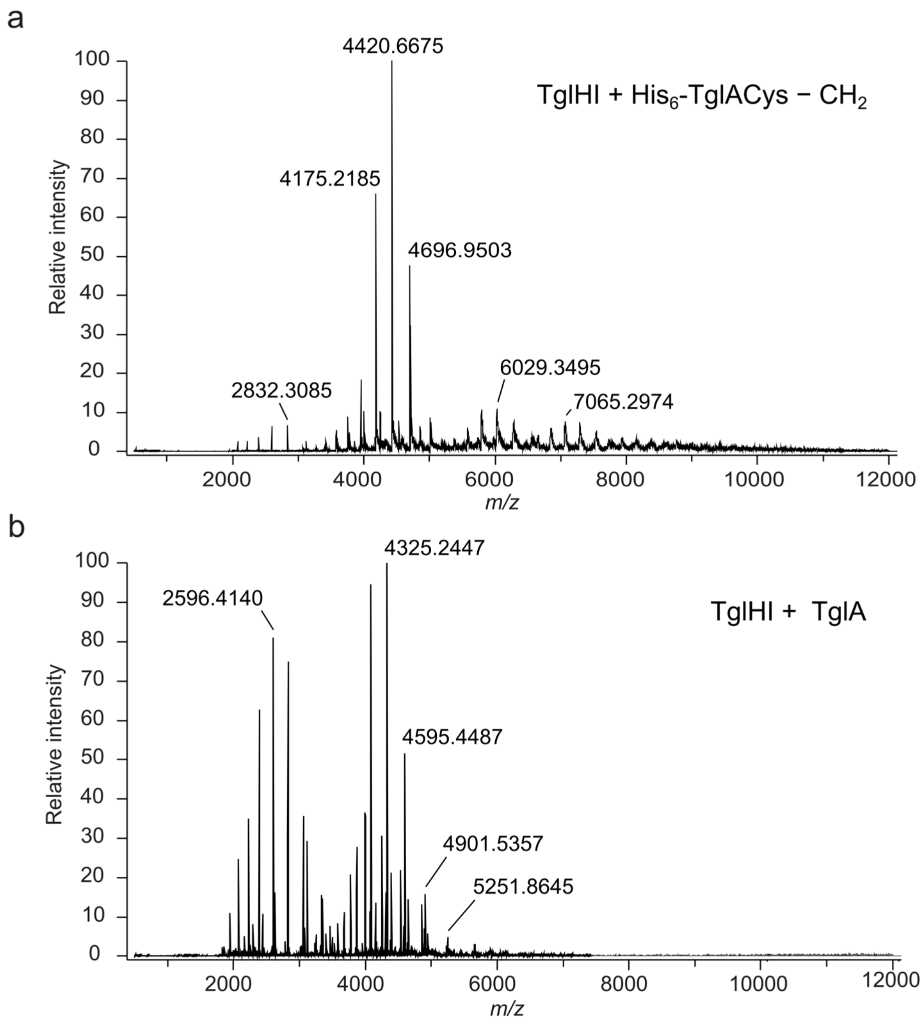

Figure S6. Native mass spectra of the TglHI-peptide complex before deconvolution. **(a)** Source spectrum of His_6-TglA-Cys – CH_2 bound to His_6-TglHI. **(b)** Source spectrum of TglA bound to His_6-TglHI. The source spectra were analysed using BioPharma Finder Software and deconvoluted using the ReSpect algorithm (see Figure 6 in the main text).

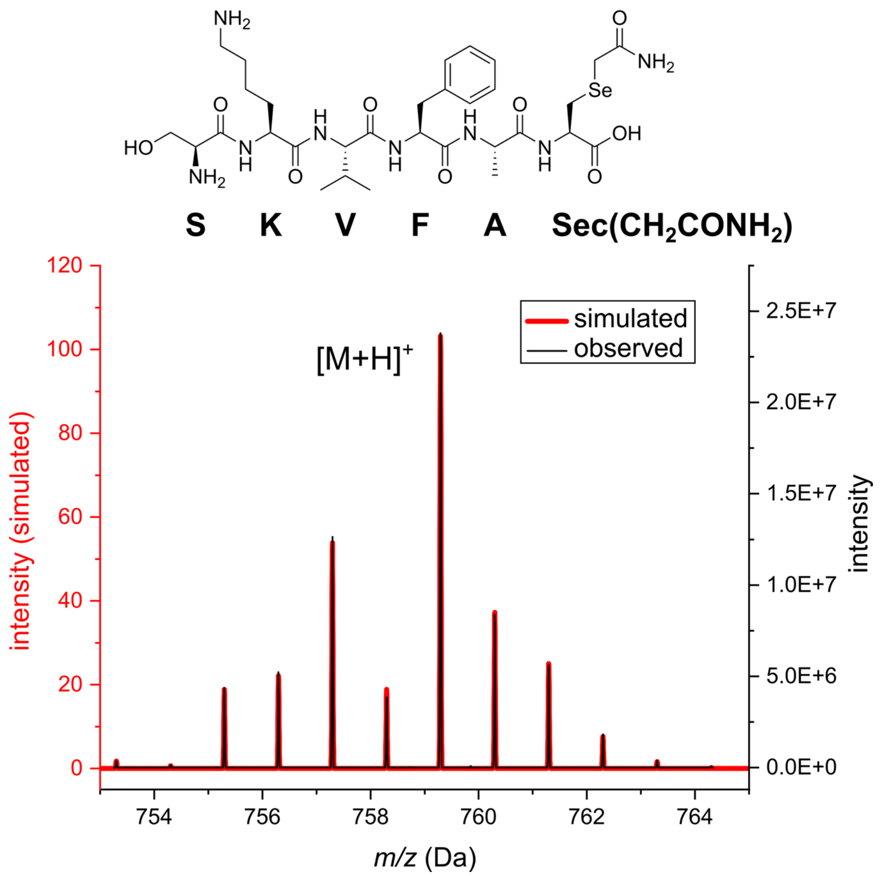

Figure S7. ESI-MS analysis of TglASec after GluC digestion and iodoacetamide alkylation. The observed $[M+H]^+$ spectrum of the Sec-containing fragment (black) is overlaid on a simulated spectrum calculated by enviPat[7] (red). The isotope distribution confirms the presence of Se in the peptide.

References

(1) Ting, C. P., Funk, M. A., Halaby, S. L., Zhang, Z., Gonen, T., and van der Donk, W. A. (2019) Use of a scaffold peptide in the biosynthesis of amino acid-derived natural products, *Science 365*, 280-284.

(2) Zhang, Z., and van der Donk, W. A. (2019) Nonribosomal peptide extension by a peptide amino-acyl tRNA ligase, *J. Am. Chem. Soc. 141*, 19625-19633.

(3) Kennedy, M. C., Kent, T. A., Emptage, M., Merkle, H., Beinert, H., and Münck, E. (1984) Evidence for the formation of a linear [3Fe-4S] cluster in partially unfolded aconitase, *J. Biol. Chem. 259*, 14463-14471.

(4) Johnson, E. C. B., and Kent, S. B. H. (2006) Insights into the mechanism and catalysis of the native chemical ligation reaction, *J. Am. Chem. Soc. 128*, 6640-6646.

(5) Jumper, J., Evans, R., Pritzel, A., Green, T., Figurnov, M., Ronneberger, O., Tunyasuvunakool, K., Bates, R., Žídek, A., Potapenko, A., Bridgland, A., Meyer, C., Kohl, S. A. A., Ballard, A. J., Cowie, A., Romera-Paredes, B., Nikolov, S., Jain, R., Adler, J., Back, T., Petersen, S., Reiman, D., Clancy, E., Zielinski, M., Steinegger, M., Pacholska, M., Berghammer, T., Bodenstein, S., Silver, D., Vinyals, O., Senior, A. W., Kavukcuoglu, K., Kohli, P., and Hassabis, D. (2021) Highly accurate protein structure prediction with AlphaFold, *Nature 596*, 583-589.

(6) Evans, R., O'Neill, M., Pritzel, A., Antropova, N., Senior, A., Green, T., Žídek, A., Bates, R., Blackwell, S., Yim, J., Ronneberger, O., Bodenstein, S., Zielinski, M., Bridgland, A., Potapenko, A., Cowie, A., Tunyasuvunakool, K., Jain, R., Clancy, E., Kohli, P., Jumper, J., and Hassabis, D. (2021) Protein complex prediction with AlphaFold-Multimer, *bioRxiv*, 2021.2010.2004.463034.

(7) Loos, M., Gerber, C., Corona, F., Hollender, J., and Singer, H. (2015) Accelerated isotope fine structure calculation using pruned transition trees, *Anal. Chem. 87*, 5738-5744.
